# Regio‐ and Stereoselective Steroid Hydroxylation at C7 by Cytochrome P450 Monooxygenase Mutants

**DOI:** 10.1002/anie.202003139

**Published:** 2020-05-25

**Authors:** Aitao Li, Carlos G. Acevedo‐Rocha, Lorenzo D'Amore, Jinfeng Chen, Yaqin Peng, Marc Garcia‐Borràs, Chenghua Gao, Jinmei Zhu, Harry Rickerby, Sílvia Osuna, Jiahai Zhou, Manfred T. Reetz

**Affiliations:** ^1^ School of life science Hubei University State Key Laboratory of Biocatalysis and Enzyme Engineering #368 Youyi Road Wuhan 430062 P.R. China; ^2^ Biosyntia ApS 2100 Copenhagen Denmark; ^3^ State Key Laboratory of Bio-organic and Natural Products Chemistry Center for Excellence in Molecular Synthesis Shanghai Institute of Organic Chemistry University of Chinese Academy of Sciences Shanghai 200032 P. R. China; ^4^ Institut de Química Computacional i Catàlisi and Departament de Química Universitat de Girona Carrer Maria Aurèlia Capmany 69 17003 Girona Catalonia Spain; ^5^ LabGenius G.01-06 Cocoa Studios 100 Drummond Rd London SE16 4DG UK; ^6^ ICREA Pg. Lluís Companys 23 08010 Barcelona Spain; ^7^ Max-Planck-Institut für Kohlenforschung Kaiser-Wilhelm-Platz 1 45470 Muelheim Germany; ^8^ Tianjin Institute of Industrial Biotechnology Chinese Academy of Sciences 32 West 7th Avenue Tianjin 300308 P. R. China

**Keywords:** cytochromes, directed evolution, enzymes, oxidation, steroids

## Abstract

Steroidal C7β alcohols and their respective esters have shown significant promise as neuroprotective and anti‐inflammatory agents to treat chronic neuronal damage like stroke, brain trauma, and cerebral ischemia. Since C7 is spatially far away from any functional groups that could direct C−H activation, these transformations are not readily accessible using modern synthetic organic techniques. Reported here are P450‐BM3 mutants that catalyze the oxidative hydroxylation of six different steroids with pronounced C7 regioselectivities and β stereoselectivities, as well as high activities. These challenging transformations were achieved by a focused mutagenesis strategy and application of a novel technology for protein library construction based on DNA assembly and USER (Uracil‐Specific Excision Reagent) cloning. Upscaling reactions enabled the purification of the respective steroidal alcohols in moderate to excellent yields. The high‐resolution X‐ray structure and molecular dynamics simulations of the best mutant unveil the origin of regio‐ and stereoselectivity.

## Graphical abstract

**One mutant, six targets**: The regio‐ and stereoselective C−H activation for hydroxylation of six different steroids with formation of C7β alcohols was accomplished using a single P450 mutant evolved by protein library construction based on a special DNA assembly and cloning procedure. The C7β steroidal alcohols, not readily accessible by synthetic reagents or catalysts, are of intense interest as therapeutic drugs.

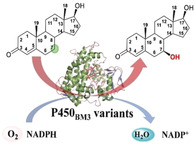

## Introduction

Ever since the landmark industrial semi‐synthesis of cortisol in the early 1950s, a synthesis which required regioselective oxidation of readily available progesterone at C11,^1^ cytochrome P450 monooxygenases (CYPs) have captured the imagination of organic chemists and biotechnologists.^2^, ^3^ One of the central challenges is the CYP‐catalyzed regio‐ and stereoselective oxidation at predetermined positions in steroids, because these complex molecules have dozens of potential sites for the placement of hydroxy functionality. Selective oxidations of steroids using modern synthetic reagents and catalysts is generally even more difficult.^4^ These hydroxysteroids have great potential as novel therapeutic drugs, with some of them already being applied.^1^, ^5^

Typically, biocatalytic steroid hydroxylation is performed using bacterial strains that can produce a desired product. However, substrate conversion is usually low because enzyme activity toward such substrates has not been optimized by natural evolution,5a the inefficiency of electron transport in multicomponent systems being one of the reasons. Moreover, it is difficult to find a microbial strain that also enables complete regio‐ and stereoselectivity. To address these problems, we have considered alternative sources. One of the most active CYPs is P450‐BM3,3b a self‐sufficient enzyme composed of a catalytic heme domain and reductase domain that contains flavins in the form of FMN and FAD for electron transfer. Using testosterone as the model substrate in two different directed evolution campaigns, we previously obtained C2β‐ and C15β‐selective mutants,^6^ and were also able to specifically achieve both C16α and C16β selectivity in a targeted manner.^7^ Rational design of other bacterial P450s has been also used in the quest to control stereo‐ and regioselectivity of steroid hydroxylation.^8^ However, to date, no general method has been devised to achieve steroid hydroxylation at any desired position in a highly regio‐ and stereoselective manner.

Traditionally, P450 mutant libraries (that were often created for other purposes) are screened with a model substrate (e.g., steroid). In these screening campaigns, if a “hit” is found exhibiting oxidative activity towards a random position, this mutant can serve as a template for subsequent steps of directed evolution to optimize protein activity and selectivity (nontargeted C−H activation). The amount of screening effort (i.e., number of samples tested), which usually involves low‐throughput HPLC analysis, dictates how successful the methodology actually is from a practical perspective. For instance, in the directed evolution examples above, 9000 bacterial colonies had to be screened for obtaining only two steroidal derivatives having either 2β‐ or 15β‐hydroxy functions.^6^ Using advanced directed evolution approaches in a subsequent study, it was nevertheless necessary to screen 3000 samples to obtain 10 different hydroxylated steroidal derivatives with either 16α‐ or 16β‐hydroxy groups.^7^

Ideally, novel protein engineering methods should enable the targeted oxidation of steroids at any desired position with high stereoselectivity and activity. However, this is still a formidable challenge that has not yet been solved in protein engineering, especially when focusing on the steroidal B ring. While working on this endeavor, we were able to engineer mutants for the nontargeted C−H activation of testosterone at position C7 with relatively low screening effort. Steroids bearing a hydroxy group at C7 have attracted particular attention, especially diastereomerically pure C7β alcohols and the respective esters, because they show significant promise as neuroprotective and anti‐inflammatory agents.^5^, ^9^ It has been stated that such compounds “can be used to protect against acute and chronic neuronal damage caused by such events as stroke, brain trauma and cerebral ischaemia such as may be induced by sub‐arachnoid haemhorrage or which occurs during heart bypass surgery, etc.”9c Moreover, recent studies have shown that bile acids bearing a hydroxy function at C7 are intimately involved in metabolic and immune processes.^10^ Unfortunately, the synthesis of these actual and potential therapeutic drugs requires tedious multistep chemical processes.1e, 1f To date, purely biocatalytic routes to steroidal C7β alcohols have also not been very expedient because of extremely low yields,1c, 5, 8 necessity of multistep enzymatic processes (not involving direct hydroxylation),1d or unfortunate formation of mixtures of regioisomers.5d In the present study, we were able to devise a simple and robust biocatalytic approach using P450‐BM3 for the biocatalytic preparation of a set of structurally different steroidal C7β alcohols.

## Results and Discussion

**Figure 1:**
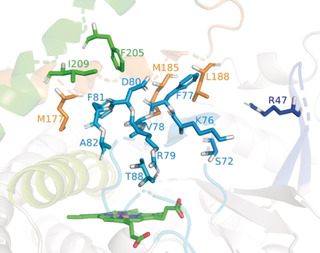
Binding pocket of P450‐BM3 triple mutant (F87G/A328G/A330W) featuring 15‐residue randomization sites: R47, S72, K76, F77, V78, R79, D80, F81, A82, T88, M177, M185, L188, F205, and I209.

Whereas wild‐type (WT) P450‐BM3 does not accept steroids with measurable activity, we discovered that the triple mutant F87G/A328G/A330W displays about 3 % selectivity towards testosterone (**1**) at position 7β (**2**; for structure see Table 1) with reasonable activity. This mutant was used as the starting template for further mutagenesis. We first considered 15 residues at or near the active site for focused mutagenesis, namely R47, S72, K76, F77, V78, R79, D80, F81, A82, T88, M177, M185, L188, F205, and I209 (Figure 1). NNK‐based saturation mutagenesis at a 15‐residue randomization site would result in a library with an astronomical (15^32^) size.^11^ Therefore, instead of creating such a library, exploratory site‐saturation mutagenesis libraries composed of 19 variants at each of the 15 sites were individually created, sequenced and screened to identify beneficial mutations involved in activity and selectivity (see the Supporting Information for details). Next, with the aim of maximizing the reshaping of the active site, a combinatorial DNA library of smaller size (relative to simultaneous randomization at 15 residues) was designed using a binary code in which the 15 residues were randomized to 2 amino acids (2^15^), including the WT and one additional one generally composed of a different side‐chain identified in the initial screening (see Table S1 in the Supporting Information). The 32,768‐member library was constructed using a new technology, developed by the biotech company LabGenius, that is based on the uracil‐specific excision reagent (USER) method.^12^ This seamless cloning method relies on the use of PCR products in which a deoxythymidine has been replaced by a deoxyuracil (dT→dU) at a position that is about 8–12 nucleotides downstream of the 5′ end.^13^ Afterwards, the PCR is treated with uracil DNA glycosidase, which excises uracil and results in 3′ overhangs that are compatible for cloning with other PCR products (DNA assembly of multiple DNA fragments) and/or the vector that undergoes a similar procedure (see Figure S1).

To construct the P450‐BM3 library, a template plasmid was provided containing the gene of interest. Figure S1 summarizes the library construction process. First, the insert was prepared from three parts. The central part was kept constant, whereas the left and right parts contained the following number of target residues for mutagenesis: 10 (namely, R47, S72, K76, F77, V78, R79, D80, F81, A82, T88) and 5 (namely, M177, M185, L188, F205 and I209), respectively. The two parts containing degeneracy were synthesized de novo as single‐stranded DNA, each containing deoxyuridine at their 5′ terminus. These single‐stranded fragments were converted into double‐stranded DNA by a single primer extension reaction. Then, the third intermediate fragment was amplified from the template plasmid with primers containing deoxyuridine. Afterwards, these three parts were assembled in a one‐pot reaction with USER‐enzyme mix and DNA ligase. In a separate reaction, the plasmid was prepared using inverse PCR with primers containing deoxyuridine. Finally, the linearized plasmid and library insert were gel purified and then assembled with USER‐enzyme mix and DNA ligase.

**Figure 2:**
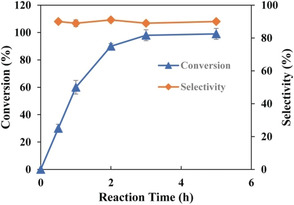
Time course of mutant LG‐23 catalyzed regio‐ and stereoselective C7β hydroxylation of **1**. Reaction conditions: 1 mm **1** in 50 mL KPi buffer pH 8.0 for 5 h, 220 rpm, 25 °C.

Next, the library was introduced and expressed in *E. coli* DE3 (Gold) using standard procedures (see the Supporting Information for details). Upon screening only 1600 variants (ca. 10 % library coverage) by automated HPLC, several improved C7β‐selective “hits” were identified (Table 1). Among them, two variants, LG‐23 and LG‐82, showed greatly improved C7β‐selectivity from 3.2 % to 82.9 % and 61.9 %, respectively. The C7α alcohol was not formed, which shows that diastereoselectivity is greater than 99 %. When the reaction using the best variant, LG‐23, was subsequently scaled up to 50 mL, substrate conversion was further improved to greater than 99 % within 5 hours and C7β selectivity increased to 90 % (Figure 2).

**Table 1:** Selected hits (improved mutants) for regio‐ and stereoselective hydroxylation of testosterone (**1**) to generate its respective C7β alcohol (**2**) found by screening a synthetic library in small scale reactions. 
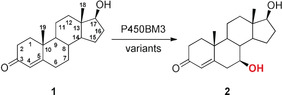

Code	Mutations^[a]^															Conv. [%]^[b]^	Selectivity [%]	
																**1**	**2**(7*β*)	Others^[c]^
	Combinatorial library																	
	R47	S72	K76	F77	V78	R79	D80	F81	A82	T88	M177	M185	L188	F205	I209			
Template																55.7	3.2	96.8
LG‐17	W	W	–	Y	L	–	–	–	L	S	–	Q	Q	–	T	66.5	9.0	91.0
LG‐22	W	–	–	Y	L	–	E	–	L	S	T	Q	–	–	T	42.0	24.6	75.4
LG‐23	W	W	–	Y	L	–	–	I	L	S	T	Q	Q	–	T	62.8	82.9	17.1
LG‐73	W	W	N	–	L	–	–	–	L	S	–	Q	Q	–	–	67.5	5.2	94.8
LG‐82	–	W	N	–	L	–	–	–	L	S	T	Q	–	I	–	31.2	61.9	38.1
LG‐109	–	W	–	–	L	–	E	–	L	S	–	L	Q	I	T	38.1	3.7	96.3

[a] Reaction conditions: 1 mm **1** in 600 μL KPi buffer pH 8.0 for 24 h, 220 rpm, 25 °C. [b] Determined by HPLC analysis; see the Supporting Information for details. [c] Other sites identified include C15β, C1β, C16β, C16α, C11α, and other unknown compounds.

We performed kinetic experiments using the purified variant LG‐23 with testosterone (**1**; see Figure S2 and Table S2). The results indicate that the mutant LG‐23 exhibits a very good catalytic performance with a *k*_cat_/K_M_ at 1.6×10^4^ m ^−1^ s^−1^ and a total turnover number of 2300±50. While these numbers indicate moderate activity compared to our previous study,^7^ it must be remembered that under upscaled conditions complete conversion within 5 hours is achieved with 90 % selectivity for the C7β product.

**Figure 3:**
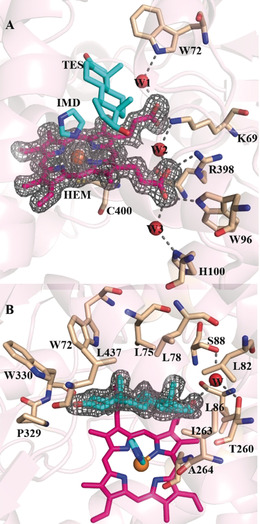
Active site of the LG‐23 mutant⋅Heme⋅**1** complex. A) Amino acids involved in heme binding are shown as a stick model in wheat, and the red spheres are water. The iron atom is shown in orange sphere. The heme (HEM), testosterone (TES) and imidazole (IMD) molecules are shown as stick model colored in magenta and cyan, respectively. 2 *F*_o_−*F*_c_ (gray mesh, contoured at 1.0 σ) electron density map for heme. Dashed lines illustrate hydrogen‐bonding interactions involving the carboxy groups of heme with residues or water. B) The residues in the testosterone‐binding site are shown as stick models in wheat, and hydrogen bonds formed between the bound testosterone and LG‐23 mutant are shown with dashed lines.

Compared to the WT enzyme, mutant LG‐23 contains 14 mutations. In an attempt to evaluate the structural basis for the selectivity of steroid binding and conversion, we carried out an X‐ray crystallographic experiment. Initial crystallization trials with the LG‐23 mutant yielded no crystals. However, when this mutant was incubated with **1** prior to crystallization, diffracting co‐crystals were obtained by the sitting drop vapor diffusion technique (see the Supporting Information for details). The complex structure of the heme‐bound LG‐23 mutant harboring **1** (LG‐23 mutant⋅Heme⋅**1**) was solved at a resolution of 1.68 Å by molecular replacement (see Table S3). The crystal belongs to space‐group C2_1_ and contains a single molecule in each asymmetric unit. The overall fold of the P450 enzyme is well‐conserved, with the heme‐binding site located at the center of an α‐helix‐rich region (see Figure S3). Carboxy groups of the heme directly make hydrogen‐bonding interactions with residues K69, W96, and R398. In addition, three water molecules (W1, W2, W3) form a continuous hydrogen‐bond network, which includes the carboxy groups of the heme, and the side chain amide of W72, K69, and H100 (Figure 3 A). Structural alignments using the Dali server^14^ confirmed that the LG‐23 mutant has highly structural similarity with P450‐BM3 mutant L86E (PDB ID:3kx3), CYP51 (PDB ID:5tl8), and CYP90B1 (PDB ID:6a17), with Z‐scores of 63.5, 36.9, and 36.5, respectively (see Figure S4).

**Figure 4:**
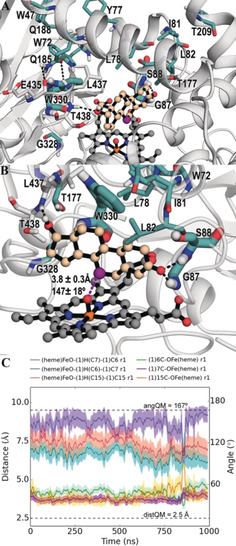
MD simulations of LG‐23 mutant. A) Representative snapshot from the MD simulations showing the active site of mutant LG‐23 and testosterone substrate bound (shown in light orange). The most important polar interactions for 7β hydroxylation are marked with a black dashed line. C7 of testosterone is highlighted in violet color. Mutated residues are shown in teal color sticks. B) Zoom of the active site of LG‐23 showing the hydrogen bond between testosterone and G87 and T438 residues (black dashed line) together with the mean value and standard deviation for the 7C⋅⋅⋅O=Fe(heme) distance and the O(Fe=O)‐(**1**)‐H(7C)‐(**1**)‐C(7) angle along the whole simulation time. C) Plot of the C6,C7,C15(**1**)⋅⋅⋅O=Fe distances (*y* primary axis) and the O(Fe=O)‐(**1**)‐H(6C,7C,15C)‐(**1**)‐C(6,7,15) angles (*y* secondary axis) along the simulation time (*x* axis) for one of the replicas (see the Supporting Information for replica 2 and 3). Ideal Quantum Mechanics (QM) distances and angles are shown with dashed lines (distQM = 2.5 Å, angQM = 167°).

Electron density of the bound substrate is of sufficient quality to allow a clear characterization of the binding modes in the LG‐23 mutant (Figure 3 B). A testosterone molecule is bound in roughly parallel orientations relative to the heme plane, with van der Waals and hydrophobic interactions to various active site residues (W72, L75, L78, L82, L86, I263, A264, P329, W330, and L437). Polar residues T260 and S88 and one water designated as **W** are also located in the substrate‐binding pocket, with S88 being responsible for hydrogen bonding with the substrate to properly position it in a catalytic pose as demonstrated by MD simulations (Figure 4).

Unfortunately, an imidazole molecule from the protein purification process is coordinated to the heme iron at the sixth position (see Figure S5). As a sequence, the distance of **1** and the iron atom in the LG‐23 mutant⋅Heme⋅**1** structure is 7.7 Å, suggesting that imidazole binding pushes **1** away from the iron atom. Therefore, the obtained X‐ray structure does not provide a fully comprehensive rationale for the improved 7β‐selectivity of the LG‐23 variant.

To investigate the molecular basis for the improved activity and 7β selectivity of the LG‐23 mutant, we performed molecular dynamics (MD) simulations with **1** bound in pose 7 (presenting C7 close to the heme), and we compared it with previous results obtained for the F87A variant.^5^, ^6^ Mutant F87A was shown to accept **1**, leading to about 20 % conversion in a similar whole‐cell system, with formation of a 1:1 mixture of 2β‐ and 15β‐hydroxytestosterone. MD simulations on the F87A variant have shown that **1** can effectively explore two different binding poses, in which the substrate is oriented alternatively with its hydroxy group towards the β1‐4 strand and the carbonyl group pointing along the I helix (pose 2, presenting the C2 carbon close to the heme) or vice versa, with the hydroxy group facing the I helix and the carbonyl towards the β1‐4 strand (pose 15, presenting C15 close to the heme).

MD simulations with **1** bound in the mutant LG‐23, performed in three independent replicas of 1000 ns each one, revealed a different binding pose that allows it to selectively undergo 7β hydroxylation (Figure 4 A and B). Indeed, in this case the substrate is placed perpendicularly with respect to pose 2 or 15, that is, the carbonyl and the hydroxy group are pointing to the B′C loop and the β4 sheet, respectively (see Figure S6). Noteworthy, in the LG‐23 mutant, the replacement of alanine by the bulkier tryptophan (A330W mutation) results in a steric clash with **1** that is reoriented along the β1‐4 strand. At the same time on the B′C loop, the mutation of phenylalanine to glycine F87G (rather than to alanine as in mutant F87A) combined with the T88S mutation, removes the steric hindrance because of the presence of the methyl groups in F87A and T88, thus allowing **1** to be oriented along the B′C loop. Our MD simulations show that in LG‐23, both G87 and S88 are involved in polar interactions with the carbonyl group of **1** [(**1**)CO⋅⋅⋅HN(G87)=2.3±0.4 Å and (**1**)CO⋅⋅⋅HN(S88)=2.6±0.5 Å mean distances, Figure 4 A,B and S7A,B], whereas on the β4 sheet, T438 interacts with the hydroxy group of **1** [(**1**)HO⋅⋅⋅HN(T438)=3.4±0.9 Å mean distance, Figure 4 A,B and S7C]. Hence, polar interactions with the B′C loop and the β4 sheet hold the substrate in pose 7, which is stable along the whole MD trajectory and shows a mean distance 7C⋅⋅⋅O=Fe(heme) of 3.8±0.3 Å, and a mean angle formed by O(Fe=O)‐(**1**)‐H(7C)‐(**1**)‐C(7) of 147±18° (see Figure S8B). These values are close to the predicted quantum mechanics (QM) ideal transition‐state geometry for H abstraction at position C7 (see Figure S8A). Remarkably, pose 7 is also the only catalytically competent pose compared to the closest adjacent C6 and C15, which show similar C⋅⋅⋅O=Fe(heme) distances but non‐optimal (heme)O‐H‐C angles to undergo C−H abstraction (Figure 4 C and S9A.B). Interestingly, the β4 sheet in mutant LG‐23 is more displaced inwards to the active site with respect to mutant F87A (see Figure S6), facilitating the interaction between T438 and the hydroxyl group of **1**. Such displacement may be triggered by the polar interactions between 1)  the amino group of Q185 and the carbonyl group of E435 ((Q185)NH⋅⋅⋅OC(E435)=4.5±2.2 Å mean distance, see Figure S7D); and 2)  the carbonyl group of Q185 and the amino group of leucine L437 ((Q185)CO⋅⋅⋅HN(L437)=2.8±1.0 Å mean distance, see Figure S7E). In fact, mutant F87A presents a methionine at position M185, whose apolar methyl sulfide group is unable to establish the same interactions and, consequently, to drag the β4 sheet inwards as mutant LG‐23 does in which M185 is mutated to glutamine (M185Q). It is worth mentioning that mutant LG‐23 includes mutations at positions 47, 72, 78, and 82, which were identified as mutational hotspots for substrate fetching and tethering in preceding studies.^6^, ^7^

**Scheme 1:**
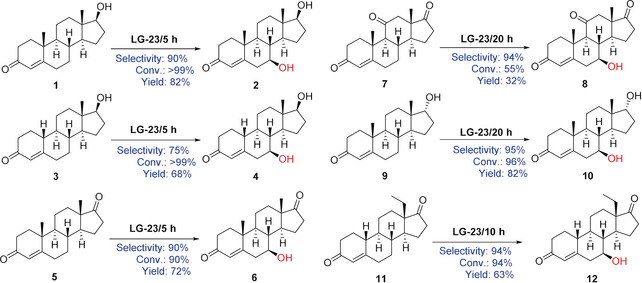
Regio‐ and stereoselective 7β hydroxylation with the mutant LG‐23 for six different steroids, testosterone (**1**), nandrolone (**3**), 4‐androstenedione (**5**), adrenosterone (**7**), epitestosterone (**9**), and d‐ethylgonendione (**11**), towards their respective C7β alcohols.

Finally, mutant LG‐23 was challenged with five different steroids in the quest to achieve high C7 regioselectivity and β‐diastereoselectivity under similar up‐scaled reaction conditions as for **1**. The substrates include nandrolone (**3**), 4‐androstenedione (**5**), adrenosterone (**7**), epitestosterone (**9**), and d‐ethylgonendione (**11**; Scheme 1; see Figures S7–S12). We found that variant LG‐23 displays excellent selectivity (90–95 %) as well as substrate conversion (90–99 %) for compounds **1**, **5**, **9**, and **11**. For **3**, mutant LG‐23 exhibited moderate selectivity (75 %), but high substrate conversion (>99 %). For **7**, LG‐23 displays slightly decreased conversion (55 %), but excellent stereoselectivity (95 %). Without optimization of the downstream processing purification step, all the C7β‐hydroxylated products were isolated with excellent purity but different yields (32–82 %). All compounds were characterized by NMR spectroscopy and LC‐MS to determine the stereoconfiguration and molecular mass (see Figures S17–S34). To the best of our knowledge, most of the steroidal C7β alcohols have not been reported to date, for example, **4**, **8**, **10**, and **12**. We note that the amount of screening in the present directed evolution campaign is notably smaller than in the sum of the previous two studies on P450‐BM3 catalyzed steroid hydroxylation.^6^, ^7^

## Conclusions

In this work, we have evolved a highly efficient P450‐BM3 mutant for the regio‐ and stereoselective oxidative hydroxylation of six different steroids with formation of the respective C7β alcohols. Instrumental for success was the design of an efficient mutagenesis strategy, supported by the use of the LabGenius platform based on DNA assembly and Uracil‐Specific Excision Reagent (USER) cloning.^12^ In principle, other DNA assembly methods can also be used as developed by alternative biotech companies and academic laboratories.^15^ In fact, the mutant library described herein could have been constructed by conventional saturation mutagenesis in our own laboratory. However, the costs of commercial preparations of such libraries are steadily decreasing, giving scientists more time to concentrate on the optimal design of focused saturation mutagenesis.^16^

The present study opens the door for pharmaceutical applications. The high‐resolution X‐ray structure flanked by MD simulations of the best mutant LG‐23 reveal how the model substrate testosterone (**1**) is constrained by the B′C loop and the β4 sheet by polar interactions, thereby enforcing the required C7β pose. The results enable new insights regarding the exquisite control of regio‐ and stereoselectivity by the evolved P450‐BM3 mutant. The lessons learned in these endeavors can be expected to be of high value in future studies directed toward P450‐BM3 catalyzed regio‐ and stereoselective oxidative hydroxylation of steroids and other substrates. Finally, we note that due to the absence of spatially close functional groups near C7 of steroids, which in principle could direct the required C−H activation, the synthesis of steroidal C7 alcohols by state‐of‐the‐art chemical methods is likely to be difficult if not impossible. The same applies to targeting other non‐activated positions in steroids, a problem that still needs to be solved in future protein engineering studies.

## Conflict of interest

The authors declare no conflict of interest.

## Supporting information

As a service to our authors and readers, this journal provides supporting information supplied by the authors. Such materials are peer reviewed and may be re‐organized for online delivery, but are not copy‐edited or typeset. Technical support issues arising from supporting information (other than missing files) should be addressed to the authors.

Supplementary
