# Supplementary material for: Regio‐ and Stereoselective Steroid Hydroxylation at C7 by Cytochrome P450 Monooxygenase Mutants
Source: Angew Chem Int Ed Engl. 2020 May 25;59(30):12499–505. doi: 10.1002/anie.202003139 (PMC7384163; doi:10.1002/anie.202003139)
Supplement: Supplementary file 1 — Supplementary [file ANIE-59-12499-s001.pdf]

Angewandte Chemie
Eine Zeitschrift der Gesellschaft Deutscher Chemiker

Supporting Information

Regio- and Stereoselective Steroid Hydroxylation at C7 by Cytochrome P450 Monooxygenase Mutants

Aitao Li[+], Carlos G. Acevedo-Rocha[+],* Lorenzo D'Amore[+], Jinfeng Chen[+], Yaqin Peng, Marc Garcia-Borràs, Chenghua Gao, Jinmei Zhu, Harry Rickerby, Sílvia Osuna,* Jiahai Zhou,* and Manfred T. Reetz**

anie_202003139_sm_miscellaneous_information.pdf

Table of contents

Materials 2

Library design 2

Screening Procedures for hydroxylation of testosterone (1) with P450-BM3 mutants 3

Scale-up reaction for preparation of 7β-hydroxylated steriods with mutant LG-23 4

Protein purification for mutant LG-23 4

Kinetics measurement of P450 BM3 mutant LG-23 5

Structural determination of LG-23 mutant complex 5

QM calculation 7

MD simulations 7

HPLC analysis 8

LC-MS analysis. 9

NMR analysis. 9

NMR data 9

Supplementary tables 12

Supplementary figure for library construction 15

Supplementary figure for kinetics 15

Supplementary figures for X-ray structures 16

Supplementary figures for computational analysis 18

HPLC choromatograms 23

LC-MS spectrums 29

NMR Spectrums 32

References 44

Materials

KOD Hot Start DNA Polymerase was obtained from Novagen. Restriction enzyme *Dpn*I was bought from New England Biolabs. The oligonucleotides were synthesized by Life Technologies. Plasmid preparation kit was ordered from Zymo Research, and PCR gel extraction kit was bought from QIAGEN. DNA sequencing was conducted by GATC Biotech. Unless otherwise indicated, all chemicals and steroid standards purchased were of the highest purity grade from Sigma-Aldrich (St. Louis, US) and Steraloids, Inc. (Newport, US), respectively. For site-directed and combinatorial saturation mutagenesis as well as protein expression, the strain *E. coli* BL21(DE3), was used (Merck-Millipore, Darmstadt, Germany), and generally cultured in lysogeny broth (LB) with 50 µg μL^{-1} kanamycin (LB^{K50}), both obtained from Carl Roth (Karlsruhe, Germany). According to standard molecular biology protocols, cells were prepared with 10% glycerol and transformed using a "MicroPulser" electroporator (BioRad, Hercules, US). Following cell recovery by addition of 1 mL LB and incubation at 37°C and 220 rpm for 1 h, whole cultures were plated onto large petri dishes containing LB^{K50} agar.

Library design

As explained in the main text, several active site residues were randomized to all 19 amino acids, followed by sequencing and screening with testosterone. The resulting screening data was used to build genotype-phenotype maps (so-called mutability landscapes) in which phenotype can be defined as percentage of testosterone conversion (i.e., activity) or selectivity to any position observed. The complete dataset targeted a total of 34 active site residues and it will be published in a separate study (Li et al. in preparation). Mutant F87G, the starting point, exhibits about 15% testosterone conversion and selectivities of 37, 21, 30 and 26% at positions 15β, 1β, 2β and others, respectively. All double mutants containing mutation gF87G and all possible amino acid combinations at several active site residues were analyzed. For example, mutations A328G and A330W exhibited increase testosterone conversion and selectivity towards position 11α. Since mutant F87G exhibited wide substrate specificities and low activity, we introduced mutation A330W resulting in about 30% testosterone conversion and 24, 48, 2, 18 and 8% selectivity towards positions 15β, 1β, 2β, 11α and others, respectively. This double mutant had a 2-fold enhancement substrate conversion and a new selectivity of 18% towards position 11α. We next used double mutant F87G/A330W (GW) and introduced mutation A328G, resulting in triple mutant F87G/A328G/A330W (GGW) exhibiting 57% testosterone conversion with 22, 28, 5, 3, 35 and 14% selectivity towards positions 15β, 1β, 2β, 7β, 11α and others, respectively. Compared to starting mutant F87G, triple mutant GGW has a 4-fold improvement of substrate conversion and new selectivity towards positions 7β and 11α with respective

values of 3 and 35 %. Instead of doing iterative site-directed mutagenesis, we decided to do a combinatorial library targeting 15 active residues that exhibited enhanced activity and/or selectivity in the initial screening using mutant F87G. The active site residues are R47, S72, K76, F77, V78, R79, D80, F81, A82, T88, M177, M185, L188, F205 and I209. A summary of the rationale behind the selection of these residues is given in Table S1. Generally, the 15 residues were randomized to the WT (in almost all cases) and an additional amino acid exhibiting enhanced activity (testosterone conversion) and/or selectivity towards positions 15β, 1β or 11α but not 7β. This is why our study can be considered as non-targeted CH activation. The choice of degenerate codons was done using the software Swiftlib[1] and the combinatorial library was prepared by Labgenius as described in the main text. The library was introduced into *E. coli* cells using standard procedures, with reasonable transformation efficiency to sample the complete library (>10,000 colonies per transformation).

Screening Procedures for hydroxylation of testosterone (1) with P450-BM3 mutants

Colonies developed on the agar plates were picked and transferred into deep-well plates containing 400 µL LB medium with 50 µg/mL kanamycin and cultured overnight at 37°C with shaking. An aliquot of 120 µL was transferred to glycerol stock plate and stored at -80 °C. The expression culture was inoculated by transfer of 100 µL overnight culture into 900 µL TB medium containing 0.2 mM IPTG and 50 µg/mL kanamycin as a final concentration. After 20 h expression at 25°C, 220 rpm, the cell pellets were harvested and washed with 400 µL 100 mM pH 8.0 potassium phosphate buffer by centrifugation at 4°C and 4000 rpm for 10 min. The supernatant was discarded and the 96-well plates were stored at -80°C until further use. When needed, libraries were thawed at room temperature (RT). Whole cells were re-suspended with 600 µL 100 mM KPi buffer (pH 8.0) containing 100 mM glucose (Applichem), 10% glycerol (Applichem), 1 mM $NADP^+$ (Merck-Millipore or Applichem), 1 U/mL glucose dehydrogenase (GDH-105) obtained from Codexis (Redwood City, US). The reaction mixtures were immediatedly frozen in liquid nitrogen. The plates were carefully thawed frist at RT for a couple of minutes, followed by a warm water bath (20-25°C) for about 30-45 min. The reaction was initiated by addition of 6 µL testosterone [stock: 100 mM (DMF); final conc. 1 mM (1%)] and incubation for 24 h at 25°C and 220 rpm with gas permeable seals. The reaction was stopped by adding 3 × 150 µL of ethyl acetate using a Tecan robotic system (Männedorf, Switzerland) equipped with a liquid handling arm (LiHA), which was controlled using Gemini software V3.50, followed by centrifugation (30 min, 4,000 rpm, 20°C). The organic phase was transferred into a multi-titer plate (MTP; Abgene, AB-0796) with the Tecan robot system. Plates were placed in the hood until complete evaporation of the solvent. The dried samples were resuspended in 150 µL acetonitrile and passed through a PTSF 96-well plate filter to remove solid particles (Pall, VWR, Germany)

into a new 500 µL MTP (Nunc). The MTP plates were closed using silicon lids and submitted to HPLC analysis.

Promissing hits were chosen and subjected to cultivation with shaking flask for further confirmation. Their DNA plasmids were extracted and submitted for sequencing to identify their mutations.

Scale-up reaction for preparation of 7β-hydroxylated steriods with mutant LG-23

The P450-BM3 mutants LG-23 was inoculated into 4 mL LB medium containing kanamycin 50 µg/mL and cultured overnight at 37°C, 220 rpm. The overnight culture (4 mL) was transferred into 200 mL TB with 50 µg/mL kanamycin in 500 mL shaking flasks. The cultivation continued at 37°C, 220 rpm for 2~3 h until the OD600 reached 0.6~0.8, then IPTG was added to a final concentration of 0.2 mM and the temperature was reduced to 25°C. After 20 h of expression, the cells were harvested and washed once with 100 mM KPi buffer (pH 8.0) by centrifugation at 4000 rpm, 4°C for 15 min. The cell pellets were stored at -80°C until further use of biotransformation. When needed for scale-up reaction, whole cells were re-suspended with 50 mL 100 mM KPi buffer (pH 8.0) containing 100 mM glucose (Applichem), 10% glycerol (Applichem), 1 mM $NADP^+$ (Merck-Millipore or Applichem), 1 U/mL glucose dehydrogenase (GDH-105) obtained from Codexis (Redwood City, US). The reaction mixtures in 50-mL falcon tubes were immediatedly frozen in liquid nitrogen. The tubes were carefully thawed frist at RT for a couple of minutes, followed by a warm water bath (20-25°C) for about 30-45 min. The reaction mixture 50 mL was then transferred into 250-mL shaking flask and the reaction was initiated by addition of 500 µL steriod (1, 3, 5, 7, 9 and 11) [stock: 100 mM (DMF); final conc. 1 mM (1%)] at 25°C and 220 rpm, the reaction process was monitored by HPLC analysis. After reaction, the 7β-hydroyxlated products were extracted with 2 × 50 mL ethyl acetate and 50 mL dichloromethane, the organic phase was then combined and dried over Na_2SO_4 overnight. The organic solvent was then evaporated and subjected to purification with flash chomatography column to obtain the purified products, which were then submitted to HPLC-MS and NMR analysis for structure identification.

Protein purification for mutant LG-23

The cell pellets were disrupted by sonication and the tube was kept in an ice bath during sonication. The collected lysate was centrifuged for 45 min at 11,000 rpm at 4°C and the obtained brownish-red supernatant was filtered to sterility with a 0.45 µm filter. The lysate obtained was loaded onto a nickel affinity column (GE Healthcare) and washed with 10~250 mM imidazole solution containing 800 mM NaCl and 50 mM potassium phosphate buffer (pH 8.0). Proteins from the flow through were pooled and concentrated, and then desalted

using Hitrap desalting column equilibrated with 100 mM potassium phosphate buffer (pH 8.0). A flow rate of 5 mL/min was used and all fractions showing adsorption at 417 nm were collected and concentrated to a final volume of 1 mL with Amicon Ultra centrifugal filters (cut off 50 kDa). The protein was shock frozen with liquid nitrogen and stored at -80°C until further usage. An aliquot was thawed at room temperature and enzyme concentration was determined by CO difference spectrum analysis prior to usage.

Kinetics measurement of P450 BM3 mutant LG-23

The test was performed using a JASCO V - 650 spectrophotometer equipped with a PAC - 743 Peltier temperature control unit and UV - Vis - NIR Spectra Manager software II. All assays were performed in 100 mM potassium phosphate buffer (pH 8.0) at 30° C using quartz cuvettes adapted for magnetic stirring (900 rpm). Kinetic parameters were determined by measuring NADPH consumption monitored at 340 nm. The NADPH regeneration system (1 mM NADPH, 10 mM glucose-6-phosphate and 2 units glucose-6-phosphate dehydrogenase) and P450-BM3 mutant LG-23 concentrations (0.25 μM) were employed in the reaction mixture. Reactions were started by adding the testosterone stock solution in DMSO with a final concentration varies from 0 mM to 1 mM. The concentration of DMSO was 1% (v/v) for all measurements. Initial reaction rates were calculated from the first 5 minutes of the reaction. Due to uncoupling reactions, where NADPH is consumed without substrate oxidation, the initial rate calculation at different concentrations of testosterone was obtained after subtraction of the initial rate of NADPH consumption in absence of substrate. K_M and k_{cat} values were estimated by plotting initial rate versus substrate concentration and fit to Michaelis-Menten using non-linear regression. For the determination of coupling efficiency using P450 mutants, consumption of NADPH was measured by NADPH depletion at 340 nm (ε = 6.22 mM^{-1} cm^{-1}) and product formation was analyzed by HPLC analysis. In a cuvette, stirred at 900 rpm, 100 mM pH=8.0 potassium phosphate was supplemented with 1 mM testosterone and 1 mM NADPH. Reaction was started with addition of 0.5 μM P450 enzyme (with a final volume of 1 mL) and monitored until absorption value for NADPH was constant (completion of the reaction). The reaction was stopped by addition of 2 x 500 μL EtOAc. The organic phase was separated by centrifugation and EtOAc phase was then subjected to GC analysis. The kinetic parameters are shown in Table S2.

Structural determination of LG-23 mutant complex

I. Over-expression and purification of LG-23 mutant for crystallization.

We expressed and purified LG-23 mutant as an N-terminal $(His)_6$ tag fused protein for crystallization. *E. coli* BL21(DE3) cells transformed with pRSFDuet-1-LG-23 mutant were grown at 37°C in 1 L of TB with kanamycin (50 mg/L) and induced by addition of 0.4 mM

IPTG (isopropyl-β-Dthiogalactopyranoside) when cell density reaches OD_{600} of 0.6. The cells continued to grow for 18 h at 25℃ and were harvested by centrifugation at 8000 rpm for 3 min at 4°C. The cell pellets were resuspended in buffer A (25 mM Tris, pH 8.0, 500 mM NaCl, 5 mM β-mercaptoethanol, 1 mM PMSF (phenylmethyl sulfonyl fluoride)), and lysed by French press with a high-pressure homogenizer (60-100 MPa) on ice. The supernatant was loaded onto a pre-equilibrated Ni-NTA affinity column (GE), washed with 100 mL buffer A, and eluted with buffer B (25 mM Tris, pH 8.0, 500 mM NaCl, 300 mM imidazole, 10 mM β-mercaptoethanol, 10% glycerol). Purified protein was concentrated using Amicon® ultra filter units with a 50 kDa molecular weight cut-off (Millipore) to yield 2 mL of protein. The concentrated protein was then applied to a HiLoad Superdex 200 column (GE Healthcare) in buffer C (25 mM Tris, pH 8.0, 150 mM NaCl, 3 mM DTT (dithiothreitol)). The fractions were collected and concentrated for crystallization.

II. Crystallization of LG-23 mutant.

Crystal structures of LG-23 mutant were obtained by the sitting drop vapor diffusion technique at 16 ºC. Purified LG-23 mutant (10 mg/mL) was incubated with 1 mM of $NADP^+$ and 2 mM of **1**) in buffer C on ice for 30 min. The protein samples were mixed in a 1:1 ratio with the reservoir solution in a 2 µL volume and equilibrated against 50 µL reservoir solution. Crystals appeared after 1~2 days at 16 ºC. The LG-23 mutant complex crystals were grown in 10% w/v PEG 20000, 20% v/v PEG MME 550, 0.03 M of each ethylene glycol (0.3 M diethyleneglycol, 0.3 M triethyleneglycol, 0.3 M tetraethyleneglycol, 0.3 M pentaethyleneglycol), 0.1 M MES/imidazole pH 6.5. Crystals were flash-frozen in liquid nitrogen with the reservoir solution directly.

III. Data Collection and Structure Determination.

The X-ray diffraction data of LG-23 mutant complex were collected at wavelength of 0.97892 Å in beamline BL19U1 at the Shanghai Synchrotron Radiation Facility (SSRF). Data reduction and integration was achieved with HKL3000[1]software package. Crystal belonged to space group $C2_1$ and the statistics for data collection are listed in Supplementary Table S3. The structure was determined by molecular replacement using Phaser with NPG-P450 BM3 crystal structure (PDB ID:4kpa) as the initial search model. Iterative cycles of model building and refinement were performed in Coot[2] and Phenix[3], respectively. Refinement statistics for each final model are recorded in Supplementary Table S3. Structure figures were drawn using PyMol[4].

QM calculation

The P450 protein-bound porphyrin complex coordinated with iron and containing an Fe=O moiety is known as compound I (cpd I). In our computational model for cpdI, substituents at the periphery of the porphyrin moiety were replaced with hydrogen atoms and the axial Fe−SCys bond in Cpd I was modeled as a Fe−SCH_3 bond. 7β-hydroxylation by p450 consists in the hydrogen atom abstraction from the 7C–H bond of the substrate testosterone (**1**) by cpdI (which is the rate limiting step), followed by radical rebound mechanism. A previous work[5] has shown the quartet C–H abstraction transition-states (TS) to be consistently lower in energy than the doublet spin states. Hence, all the calculations were performed in the quartet state under the unrestricted formalism. In order to obtain the optimal geometry for the TS, a comprehensive computational DFT study was performed with the Gaussian09(D.01)[6] program package using the B3LYP[7,8] hybrid functional and LANL2DZ pseudopotential with associated basis set[9] to describe the Fe atom and relativistic effects. All the other atoms were treated with a 6-31g(d) basis set for the transition state optimization and subsequent frequency calculation. The Grimme's D_3 formalism[10] was used in all the calculations to correct the dispersion energy.

MD simulations

Long-timescale Molecular Dynamics simulations (MD) in explicit water were performed using AMBER 16 package[11] in our in-house GPU cluster Galatea. Substrate (**1**) parameters for the MD simulations were generated within the antechamber module of AMBER 16 using the general AMBER force field (GAFF) [12], with partial charges set to fit the electrostatic potential generated at the HF/6-31G(d) level by the restrained electrostatic potential (RESP) model[13]. The charges were calculated according to the Merz-Singh-Kollman scheme[14,15] using the Gaussian09(D.01)[6] program package. Parameters for the haem compound I and the axial Cys were taken from ref. [16]. Amino acid protonation states were predicted using the H++ server (http://biophysics.cs.vt.edu/H++)[17]. Then, the enzyme was solvated in a pre-equilibrated truncated hexagonal box with a 10-Å buffer of TIP3P[18] water molecules using the AMBER16 leap module, resulting in the addition of ~13,000 solvent molecules. The systems were neutralized by addition of explicit counterions (Na+ and Cl−). All subsequent calculations were done using the widely tested Lindorff-Larsen modification of the Amber99 force field (ff99SBildn) [19]. Due to the lack of X-ray structure at the start of the project, the structure of the LG-23 mutant used in the MD simulations was generated from the P450-BM3 original variant (pdb ID: 1FAG)[20], by removal of the palmitoleic acid and further introduction of the corresponding mutations via the RosettaBackrub web-server (https://kortemmeweb.ucsf.edu/backrub)[21-23]. Comparison of the most stable conformation after extensive MD simulations and the available X-ray data reveal almost identical

structures (see Figure S6 below). The substrate-bound structure pose 7 was obtained by manual docking of **1** with its C7 atom towards the haem-moiety. The stability of the aforementioned pose was then tested with MD simulations. A two-stage geometry optimization approach was performed. The first stage minimizes the positions of solvent molecules and ions imposing positional restraints on solute by a harmonic potential with a force constant of 500 kcal mol^{-1} $Å^{-2}$, and the second stage is an unrestrained minimization of all the atoms in the simulation cell. The systems are gently heated using six 50-ps steps, incrementing the temperature 50 K each step (0–300 K) under constant volume and periodic boundary conditions. Water molecules were treated with the SHAKE algorithm such that the angle between the hydrogen atoms is kept fixed. Long-range electrostatic effects were modeled using the particle-mesh-Ewald method[24]. An 8-Å cutoff was applied to Lennard-Jones and electrostatic interactions. Harmonic restraints of 10 kcal/mol were applied to the solute, and the Langevin equilibration scheme was used to control and equalize the temperature. The time step was kept at 1 fs during the heating stages, allowing potential inhomogeneities to self-adjust. Each system was then equilibrated without restrains for 2 ns with a 2-fs timestep at a constant pressure of 1 atm and temperature of 300 K. After the systems were equilibrated in the NPT ensemble, for both apo and substrate-bound structures, three independent 1 µs MD simulations (i.e. 3 µs accumulated) were performed under the NVT ensemble and periodic-boundary conditions using our Galatea cluster (composed by 178 GTX1080 GPUs). With Galatea, simulations for these systems were performed at a speed of ca. 100 ns/day.

HPLC analysis

The automated Shimadzu LC-2010 HPLC system (Japan) equipped with four MTP racks was used by employing a reverse-phase “250 Eclipse XDB” C18 column of 250 mm (1.8 µM size particle) together with a corresponding pre-column bought from Agilent (Waldbronn, Germany) as stationary phase. The mobile phase consisted of a mixture of high-purity water generated from the local deionized water supply using a TKA MicroLab water purification system, acetonitrile (CH_3CN) and methanol (MeOH). For all steriods (1, 3, 5, 7 and 9), a gradient program of 8 min, 1.5 mL/min flow rate and 40° C oven temperature, was used (CH_3CN:MeOH:H_2O): 0 →3 min (15:15:70), 3 → 5 min (20:20:60), 5 → 6 min (30:30:40), 6 → 7 min (15:15:70). For all steriod substrates, this protocol allows the separation of >14 known oxidation products. For 13-Ethylgon-4-ene-3,17-dione (11), a gradient program of 20 min, 1.5 mL/min flow rate and 40° C oven temperature, was used (CH_3CN:MeOH:H_2O): 1 min (15:15:70), 1 → 2 min (20:20:60), 2 → 17 min (40:40:20), 17 → 20 min (15:15:70). The corresponding HPLC chromatograms are shown in Figure S7-12.

LC-MS analysis.

For Liquid Chromatography-Mass Spectrometry (LC-MS), we used a Shimadzu LC20 XR system equipped with 3 LC20-AD XR pumps for performing a ternary gradient. Injection was performed by a SIL-20AC XR autosampler. The temperature was controlled by a CTO-20AC column oven (35°C). UV-detection was performed with a diode array detector SPD-M20A (UV at 245 nm) and the high-resolution masses were measured with the LCMS-IT-TOF mass spectrometer. The system is controlled via a CBM-20A controller and results calculation was performed with the Shimadzu LCMS Solution software, Version 3.70.390. Analytical conditions: A Column of 50 mm Poroshell 120 EC-C18, 3.0 mm i.d., 2.7 µm (Agilent Technologies) was used with a mobile phase composed of water (A), methanol (B) and acetonitrile (C). We used a linear gradient of 80% A, 10% B and 10% C in 5 min to 60% A, 30% B, and 30% C.

NMR analysis.

All Nuclear Magnetic Resonance (NMR) spectra were recorded on an Avance III 500 spectrometer (499.89MHz) equipped with a $BBFO_{plus}$ ^{1}H/BB(incl. ^{19}F) probehead with z-gradient or on an AVANCE I 600 MHz Spectrometer equipped with a cryogenically-cooled TCI probehead with z-gradients, both from Bruker Biospin GmbH. All NMR data were processed and analysed with Topspin 3.2 (Bruker). Full ^{1}H and ^{13}C assignments, including the stereospecific assignment of prochiral ^{1}H were obtained at 25°C from standard 1D experiments as well as 2D correlation experiments. The correlation experiments included ^{1}H, ^{1}H DQF-COSY, ^{1}H,^{1}H-NOESY, ^{1}H,^{13}C-HSQC, ^{1}H,^{13}C-HMBC. The ^{1}H and ^{13}C chemical shifts were referenced against TMS whereas the Ξ values corresponding to TMS were applied for ^{15}N (7.226317%), ^{31}P (40.480742%) and ^{19}F (94.094011%).

NMR data

7β-Hydroxytestosterone (2) (12.5 mg, yield 82%)

^{1}H NMR (500 MHz, Chloroform-*d*) δ 5.75 (dt, *J* = 2.0, 1.0, 0.6 Hz, 1H, 4), 3.63 (t, *J* = 9.2, 8.3 Hz, 1H, 17), 3.45 (ddd, *J* = 11.2, 9.5, 5.2 Hz, 1H, 7), 2.53 (ddd, *J* = 14.0, 5.2, 0.6 Hz, 1H, 6eq), 2.43 (ddd, *J* = 14.0, 11.2, 2.0 Hz, 1H, 6ax), 2.42 (ddd, *J* = 16.9, 14.4, 5.0 Hz, 1H, 2ax), 2.34 (dddd, *J* = 16.9, 4.9, 3.2, 1.0 Hz, 1H, 2eq), 2.09 (dtd, *J* = 13.6, 9.6, 9.2, 5.9 Hz, 1H, 16α), 2.04 (ddd, *J* = 13.5, 5.0, 3.2 Hz, 1H, 1eq), 1.88 (dddd, *J* = 12.7, 9.6, 7.4, 3.6 Hz, 1H, 15α), 1.87 (dt, *J* = 12.7, 4.1, 2.8 Hz, 1H, 12eq), 1.72 (qd, *J* = 12.7, 12.2, 5.9 Hz, 1H, 15β), 1.70 – 1.62 (m, 1H, 11eq), 1.69 – 1.60 (m, 1H, 1ax), 1.63 (q, *J* = 10.9, 9.5 Hz, 1H, 8), 1.49 (tdd, *J* = 13.6, 12.2, 8.3, 3.6 Hz, 1H, 16β), 1.46 (qd, *J* = 13.6, 12.5, 4.1 Hz, 1H, 11ax), 1.22 (d, *J* = 0.7 Hz, 3H, 19), 1.15 (ddd, *J* = 12.2, 10.6, 7.4 Hz, 1H, 14), 1.07 (tdq, *J* = 13.3, 12.7, 4.3, 0.7 Hz, 1H, 12ax), 0.93 (ddd, *J* = 12.5, 10.9, 4.2 Hz, 1H, 9), 0.81 (d, *J* = 0.7 Hz, 3H, 18).

^{13}C NMR (126 MHz, $CDCl_3$) δ 199.44 (3), 167.55 (5), 124.90 (4), 81.27 (17), 75.11 (7), 50.89 (9), 50.09 (14), 43.70 (13), 43.26 (8), 42.43 (6), 38.21 (10), 36.48 (12), 35.85 (1), 34.09 (2), 30.92 (16), 26.52 (15), 20.75 (11), 17.50 (19), 11.28 (18).

7β-Hydroxynandrolone (4) (10.0 mg, yield 68%)

^{1}H NMR (500 MHz, Chloroform-*d*) δ 5.85 (t, *J* = 2.2 Hz, 1H, 4), 3.64 (t, *J* = 8.7 Hz, 1H, 17), 3.48 (td, *J* = 11.1, 9.5, 4.8 Hz, 1H, 7), 2.71 (dd, *J* = 14.0, 4.8 Hz, 1H, 6eq), 2.44 – 2.37 (m, 1H, 2eq), 2.32 – 2.25 (m, 1H, 6ax), 2.32 – 2.25 (m, 1H, 1eq), 2.30 – 2.23 (m, 1H, 2ax), 2.14 – 2.04 (m, 1H, 16α), 2.09 – 2.04 (m, 1H, 10), 1.94 – 1.89 (m, 1H, 11eq), 1.90 – 1.86 (m, 1H, 15α), 1.88 – 1.83 (m, 1H, 12eq), 1.72 (qd, *J* = 12.4, 5.9 Hz, 1H, 15β), 1.53 – 1.47 (m, 1H, 1ax), 1.51 – 1.48 (m, 1H, 16β), 1.43 (q, *J* = 10.9, 10.5, 9.5 Hz, 1H, 8), 1.38 – 1.27 (m, 1H, 11ax), 1.18 (td, *J* = 12.2, 10.9, 7.4 Hz, 1H, 14), 1.09 (td, *J* = 13.0, 3.9 Hz, 1H, 12ax), 0.91 – 0.80 (m, 1H, 9), 0.82 (s, 3H, 18).

^{13}C NMR (126 MHz, $CDCl_3$) δ 199.88, 163.22, 125.49 (4), 81.30 (17), 74.34 (7), 49.31 (14), 47.57 (8), 46.64 (9), 45.13 (6), 43.87 (13), 41.79 (10), 36.53 (2), 36.46 (12), 30.85 (16), 26.76 (1), 26.31 (15), 26.03 (11), 11.26 (18).

7β-Hydroxy-4-androstenedione (6) (10.8 mg, yield 72%)

^{1}H NMR (500 MHz, Chloroform-*d*) δ 5.77 (t, *J* = 2.0, 1.0 Hz, 1H, 4), 3.57 (ddd, *J* = 11.1, 9.5, 5.2 Hz, 1H, 7), 2.57 (dd, *J* = 13.9, 5.2 Hz, 1H, 6eq), 2.48 (ddd, *J* = 13.9, 11.1, 2.0 Hz, 1H, 6ax), 2.46 (ddd, *J* = 19.4, 8.9, 1.2 Hz, 1H, 16β), 2.42 (ddd, *J* = 17.0, 14.2, 5.0 Hz, 1H, 2ax), 2.36 (dddd, *J* = 17.0, 5.0, 3.3, 1.0 Hz, 1H, 2eq), 2.31 (dddd, *J* = 12.8, 8.9, 6.0, 1.2 Hz, 1H, 15α), 2.10 (dt, *J* = 19.4, 9.5, 8.9 Hz, 1H, 16α), 2.05 (ddd, *J* = 13.4, 5.0, 3.3 Hz, 1H, 1eq), 1.93 (tt, *J* = 12.8, 9.5, 8.9 Hz, 1H, 15β), 1.86 (dt, *J* = 13.2, 4.1, 2.7 Hz, 1H, 12eq), 1.77 (q, *J* = 10.9, 10.5, 9.5 Hz, 1H, 8), 1.73 (dq, *J* = 13.7, 4.1, 2.7 Hz, 1H, 11eq), 1.66 (td, *J* = 14.2, 13.4, 5.0 Hz, 1H, 1ax), 1.49 (qd, *J* = 13.7, 12.5, 4.1 Hz, 1H, 11ax), 1.47 (ddd, *J* = 12.5, 10.5, 6.0 Hz, 1H, 14), 1.25 (td, *J* = 13.5, 4.1 Hz, 1H, 12ax), 1.23 (d, *J* = 0.7 Hz, 3H, 19), 1.00 (td, *J* = 12.5, 10.9, 4.1 Hz, 1H, 9), 0.94 (d, *J* = 0.6 Hz, 3H, 18).

^{13}C NMR (126 MHz, $CDCl_3$) δ 220.79 (17), 199.30 (3), 166.88 (5), 125.11 (4), 74.40 (7), 50.89 (9), 50.64 (14), 48.16 (13), 42.83 (6), 42.63 (8), 38.18 (10), 36.06 (16), 35.74 (1), 34.00 (2), 31.33 (12), 25.09 (15), 20.49 (11), 17.49 (19), 14.08 (18).

7β-Hydroxyandrenosterone (8) (5.0 mg, yield 32%)

^{1}H NMR (500 MHz, Chloroform-*d*) δ 5.76 (br s, 1H), 3.74 (ddd, *J* = 10.9, 8.8, 5.4 Hz, 1H), 2.76 (ddd, *J* = 13.7, 5.1, 3.3 Hz, 1H), 2.60 – 2.41 (m, 6H, 6a, 16b, 6b, 15a, 12b, 2b), 2.36 – 2.23 (m, 3H,2a, 12a, 16a), 2.14 – 1.99 (m, 4H, 8, 14, 15b), 1.95 (d, *J* = 10.6 Hz, 1H), 1.65 – 1.52 (m, 1H, 1a), 1.47 (s, 3H, 19), 0.90 (br s, *J* = 0.8 Hz, 3H, 18).

^{13}C NMR (126 MHz, $CDCl_3$) δ 217.42 (17), 207.05 (11), 199.54 (3), 164.40 (5), 125.74 (4), 74.14 (7), 60.70 (9), 50.89 (13), 50.40 (12), 49.81 (14), 43.36 (8), 42.49 (6), 37.90 (10), 36.37 (16), 34.65 (1), 33.79 (2), 32.07, 24.87 (15), 17.51 (19), 14.93 (18).

7β-Hydroxyepitestosterone (10) (12.3 mg, yield 82%)

^{1}H NMR (500 MHz, Chloroform-*d*) δ 5.77 (d, *J* = 2.0 Hz, 1H, 4), 3.72 (d, *J* = 6.0 Hz, 1H, 17), 3.49 (ddd, *J* = 11.3, 8.7, 5.1 Hz, 1H, 7), 2.54 (dd, *J* = 14.0, 5.1 Hz, 1H, 6eq), 2.44 (ddd, *J* = 14.0, 11.3, 2.0 Hz, 1H, 6ax), 2.43 (ddd, *J* = 16.9, 14.4, 5.0 Hz, 1H, 2ax), 2.35 (dddd, *J* = 16.9, 4.9, 3.2, 1.0 Hz, 1H, 2eq), 2.26 – 2.16 (m, 1H, 16β), 2.05 (dt, *J* = 13.5, 5.0, 3.2 Hz, 1H, 1eq), 2.04 (d, *J* = 11.9 Hz, 1H, 15α), 1.77 – 1.45 (m, 9H, 11eq, 1ax, 8, 12ax, 15b,14, 12eq, 16a, 11ax), 1.22 (s, 3H, 19), 1.03 – 0.94 (m, 1H, 9), 0.73 (s, 3H, 18).

^{13}C NMR (126 MHz, $CDCl_3$) δ 199.53 (3), 167.80 (5), 124.89 (4), 78.74 (17), 75.12 (7), 50.67 (9), 47.49 (14), 46.14 (13), 43.46 (8), 42.29 (6), 38.21 (10), 35.86 (1), 34.08 (2), 32.91 (16), 31.14 (12), 20.60 (11).

7β-13-Ethylgon-4-ene-3,17-dion (12). (9.5 mg, yield 62%)

^{1}H NMR (400MHz, Chloroform-*d*) 5.38 (s, 1H, 3), 3.55 (m, 1H, 8), 2.69 (m,1H,1), 2.18~2.41 (m, 7H, 1, 7, 13, 15, 16), 1.85~2.08 (m, 4H, 5, 6, 13, 15), 1.33-1.64 (m, 5H, 11, 14, 19), 1.19~1.21 (m, 2H, 6, 10), 1.03 (m, 1H, 9), 0.73 (t, J=7.5Hz, 3H, 19).

^{13}C NMR (101MHz, Chloroform-*d*) 219.29 (17), 199.42 (2), 162.18 (4), 125.59 (3), 73.69 (8), 51.13 (12), 50.55 (9), 46.48 (10), 46.38 (5), 45.39 (11), 41.54 (7), 36.85 (1), 36.33 (16), 26.83 (13), 26.56 (6), 25.13 (14), 24.20 (15), 17.67 (19),7.35 (20).

Supplementary tables

Table S1. Library design based on parent mutant F87G, while the library was constructed using triple mutant F87G/A328G/A330W as template. The 15 residues were randomized to the WT (in almost all cases) and an additional amino acid exhibiting enhanced activity (testosterone conversion) and/or selectivity at one or more different positions. The values indicated are relative to those observed in mutant F87G, which shows about 15% testosterone conversion and selectivities of 37, 21, 30 and 26% at positions 15β, 1β, 2β and others, respectively.

Target Residue	Degenerate Codon	Binary code for randomization	Rationale
R47	WGG	R	WT
		W	Activity enhancement (26 %)
S72	TSG	S	WT
		W	Activity enhancement (35 %)
K76	AAM	K	WT
		N	15-β selectivity (6 %); 11-α selectivity (2 %)
F77	TWC	F	WT
		Y	1-β selectivity (5 %)
V78	STA	V	WT
		L	Activity enhancement (20 %); 15-β selectivity[25]
R79	ASA	R	WT
		T	15-α selectivity (3 %); 4 selectivity (5 %); 11-α selectivity (2 %)
D80	GAM	D	WT
		E	Activity enhancement (13 %)
F81	WTC	F	WT
		I	4 selectivity (4 %)
A82	CWA	L	Activity enhancement (13 %)
		Q	1-β selectivity (30 %)
T88	TYA	S	Similar to WT (deg. codon); 15-α selectivity (3 %)
		L	11-α selectivity (12 %); 1-β selectivity (21 %); 15-α selectivity (2 %)
M177	AYG	M	WT
		T	Activity enhancement (25 %); 15-α selectivity (13 %)
M185	CWA	L	Similar to WT (deg. codon)
		Q	Activity enhancement (46 %)
L188	CWA	L	WT
		Q	Activity enhancement (21 %)
F205	WTC	F	WT
		I	Activity enhancement (4 %); 15-β selectivity (5 %)
I209	AYA	I	WT
		T	1-β selectivity (4 %)

Table S2. Coupling efficiency of P450BM3 LG-23 catalyzed 7β-hydroxylation of testosterone. [a]

**Catalyst**	NADPH consumption rate in absence of substrate (μmol/μmol*min)	NADPH consumption rate (μmol/μmol*min)	Coupling efficiency(%)[b]
LG-23	932 ± 50	824 ± 50	4.0

[a]Reaction conditions: 0.5 μM enzyme, 1 mM testosterone, 1 mM NADPH. Reaction mixtures were incubated at 750 rpm at 30 °C for 5 minutes.

[b] Coupling efficiency was calculated as the amount of the product produced divided by the amount of NADPH consumpted.

Table S3. Data collection and refinement statistics.

	LG-23 mutant•Heme•1
**Data collection**	
**Space group**	$C2_1$
**Cell dimensions**	
**a, b, c (Å)**	96.54, 55.74, 94.40
**α, β, γ (˚)**	90.00, 94.33, 90.00
**Wavelength (Å)**	0.97892
**Resolution (Å)**	50.00-1.68 (1.71-1.68)
R_{merge}	0.057(0.905)
**I/σI**	34.294(1.889)
**Completeness (%)**	100.0(99.7)
**Redundancy**	6.7(6.0)
**CC1/2**	0.720(0.715)
**Refinement**	
**Resolution (Å)**	41.60-1.68
**No. reflections**	55232
R_{work} / R_{free}	0.159/0.193
**Number of atoms**	
**Protein**	3688
**Ligand**	69
**Water**	402
***B*-factors ($Å^2$)**	
**Protein**	20.81
**Ligand**	20.99
**R.m.s. deviations**	
**bond lengths (Å)**	0.011
**bond angles (˚)**	1.259
**Ramachandran (%)**	
**outliers**	0.00
**favored**	97.53
**allowed**	2.47
**PDB code**	**6LY4**

*Values in parentheses are for highest-resolution shell.

Supplementary figure for library construction

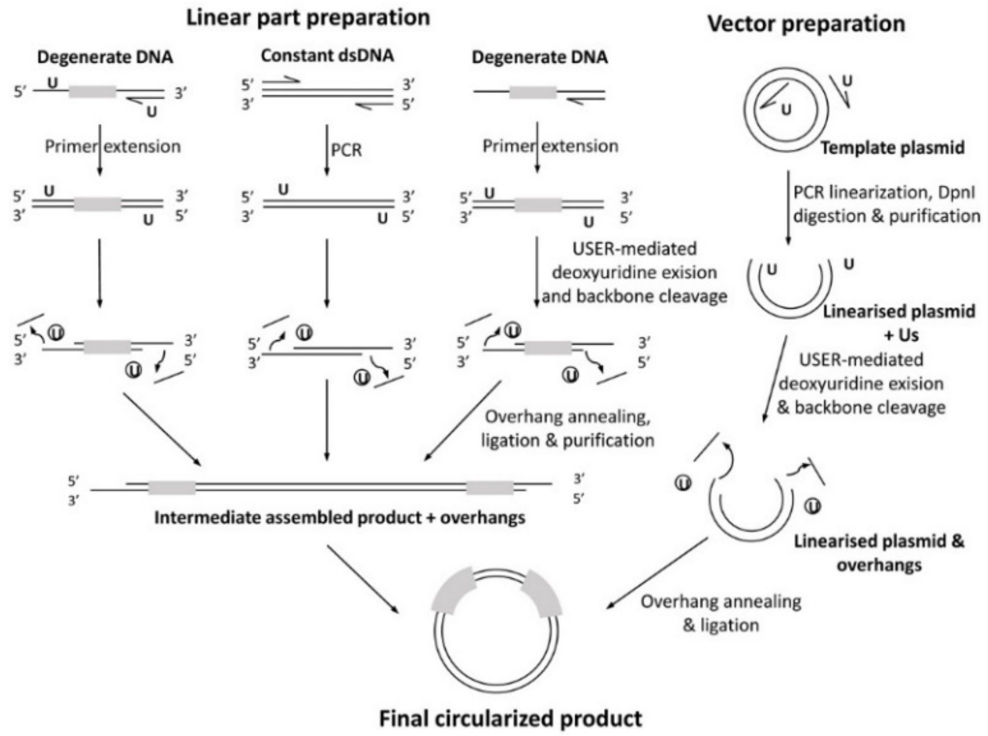

Figure S1. Schematic illustration of the USER-based combinatorial library construction by the biotech company LabGenius.

Supplementary figure for kinetics

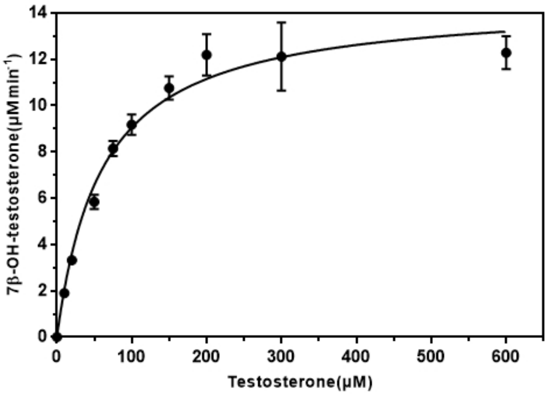

Figure S2. Steady-state kinetic behavior of evolved P450BM3 mutants LG-23. Conditions: 0.25 μM enzyme, testosterone (0-1 mM), NADPH regeneration system (1 mM NADPH, 10 mM glucose-6-phosphate and 2 units glucose-6-phosphate dehydrogenase). Reactions were allowed to proceed over a variety of timescales between 0 and 5 minutes with shaking at 750 rpm at 30 °C.

Supplementary figures for X-ray structures

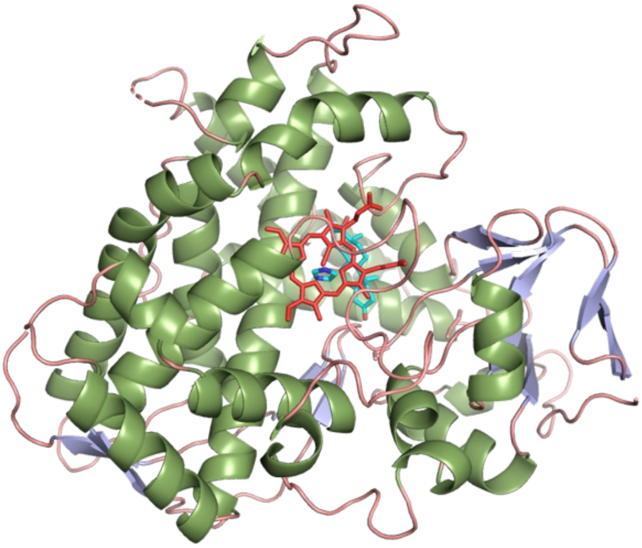

Figure S3. The overall structure is shown in cartoon model. The α-helices and β-strands are represented in smudge and light blue, respectively. The heme (HEM), testosterone (TES) and imidazole (IMD) molecules are shown as stick model colored in magenta and cyan, respectively. The structure of LG-23 mutant•Heme•1 adopts the characteristic triangular P450-fold and contains 19 α-helices and11-β strands. And the thiol group of C400 coordinates the central iron of heme on its proximal side

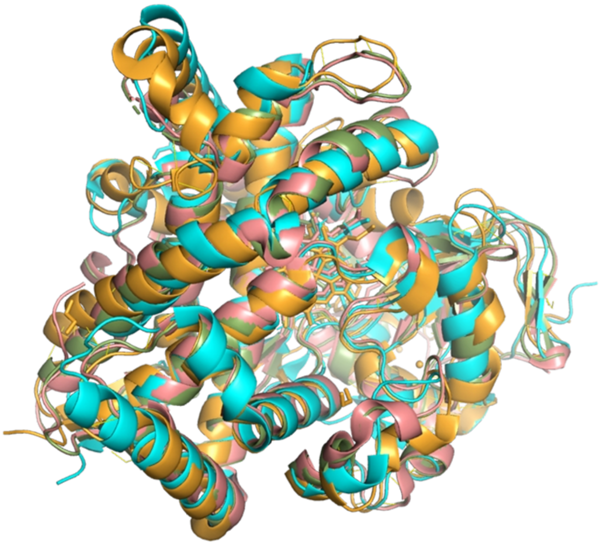

Figure S4. Superposition of LG-23 mutant•Heme•1 (smudge), P450 BM3 mutant L86E(salmon), CYP51(bright orange) and CYP90B1(cyan). The RMSD of Cα-backbone atoms is 0.311 Å for 393 atoms, 2.683 Å for 300 atoms and 1.331 Å for 153 atoms, respectively.

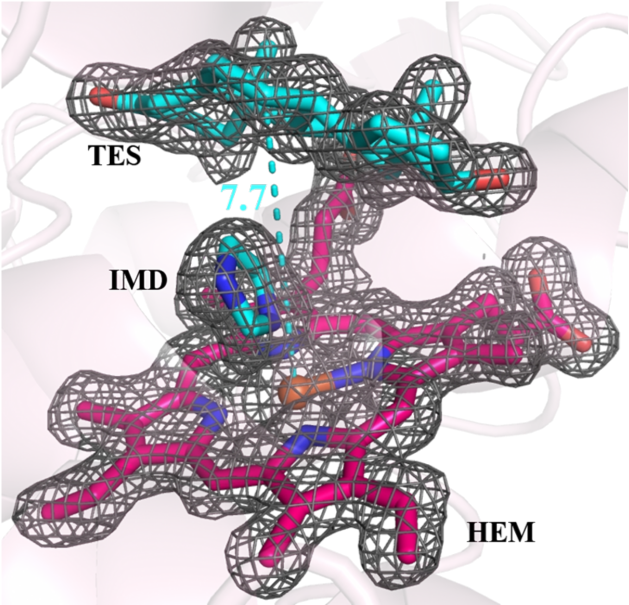

Figure S5. Binding sites of LG-23 mutant•Heme•1. 2Fo-Fc (gray mesh, contoured at 1.0 σ) electron density map for heme, TES and IMD.

Supplementary figures for computational analysis

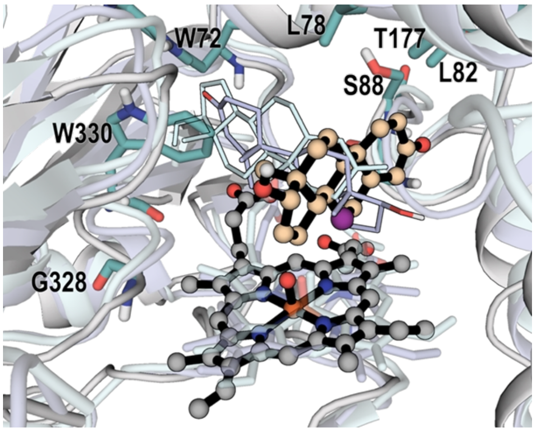

Figure S6. Overlay of representative snapshots from the MD simulations of mutant LG-23 with **1** bound in pose 7 (black sticks and light orange spheres) and mutant F87A with **1** bound in pose 2 (pale cyan sticks) and pose 15 (violet sticks). In mutant LG23 pose7, the 7C atom of **1** is highlighted in violet color and the mutated residues are shown in teal color sticks.

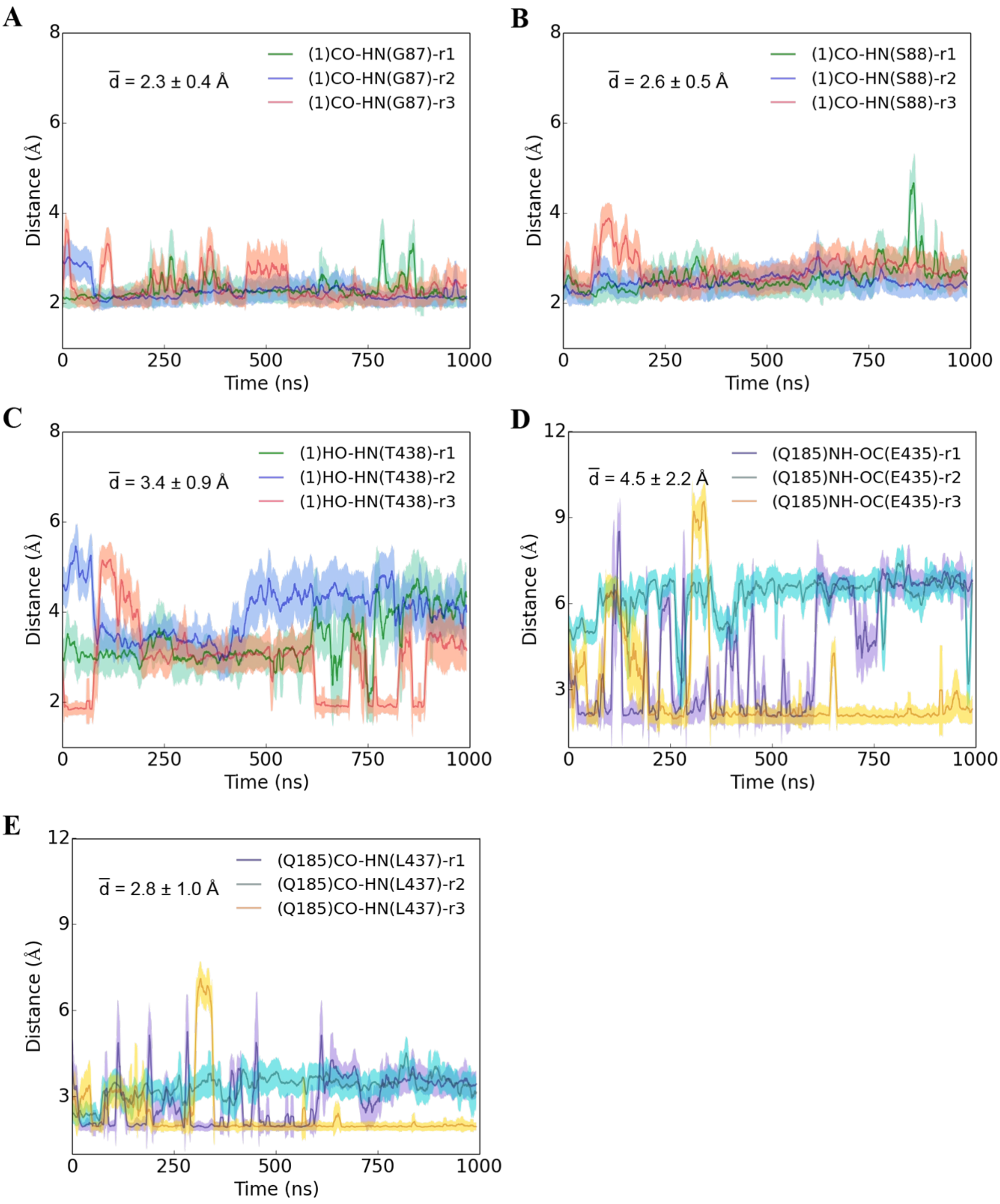

Figure S7. Plot of the distances (A) (**1**)CO···HN(G87); (B) (**1**)CO···HN(S88); (C) (**1**)CO···HN(T438); (D) (Q185)NH···OC(E435); (E) (Q185)CO···HN(L437) for the three MD replica dataset. Mean values (in Å) and standard deviations for the whole MD replica dataset are shown.

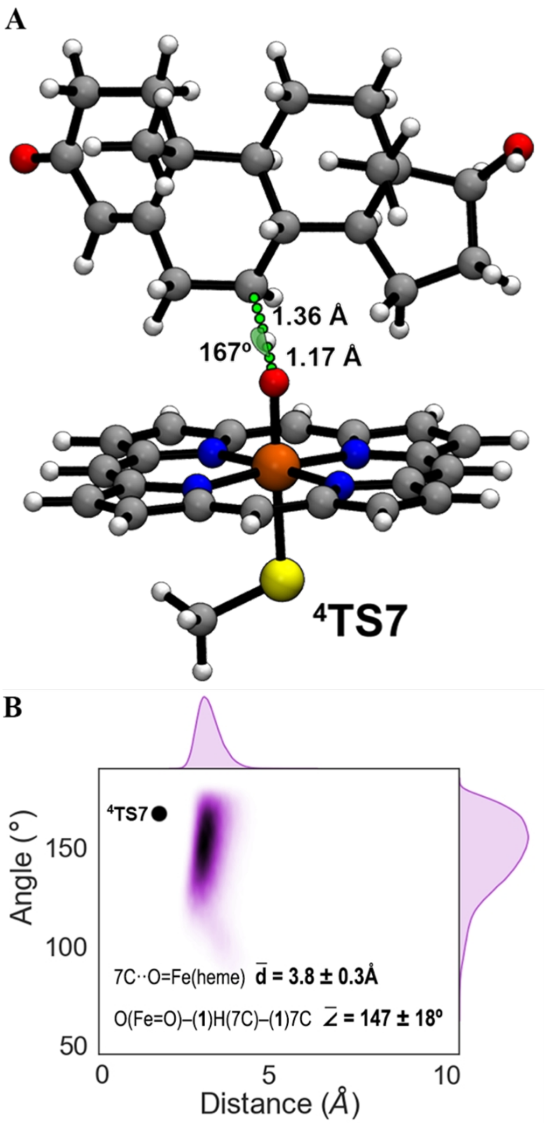

Figure S8. (A) Transition State (TS) and geometrical parameters for the C(7)-H abstraction in **1** along the quartet energy surface. (B) Distances determined between the oxygen atom of the Fe=O and the C7 atom of **1** (x axis) and angles formed by O(Fe=O) – (**1**)-H(C7) – (**1**)-C(7) (y axis) along the whole simulation time (3 joint MD replicas). The ideal distance and angle for the ^{4}TS (black dot) corresponds to the Density Functional Theory (DFT) optimized geometry for the 7C–H abstraction by CpdI using a truncated computational model. Mean distance (in Å), mean angle (in degrees) and corresponding standard deviations are shown.

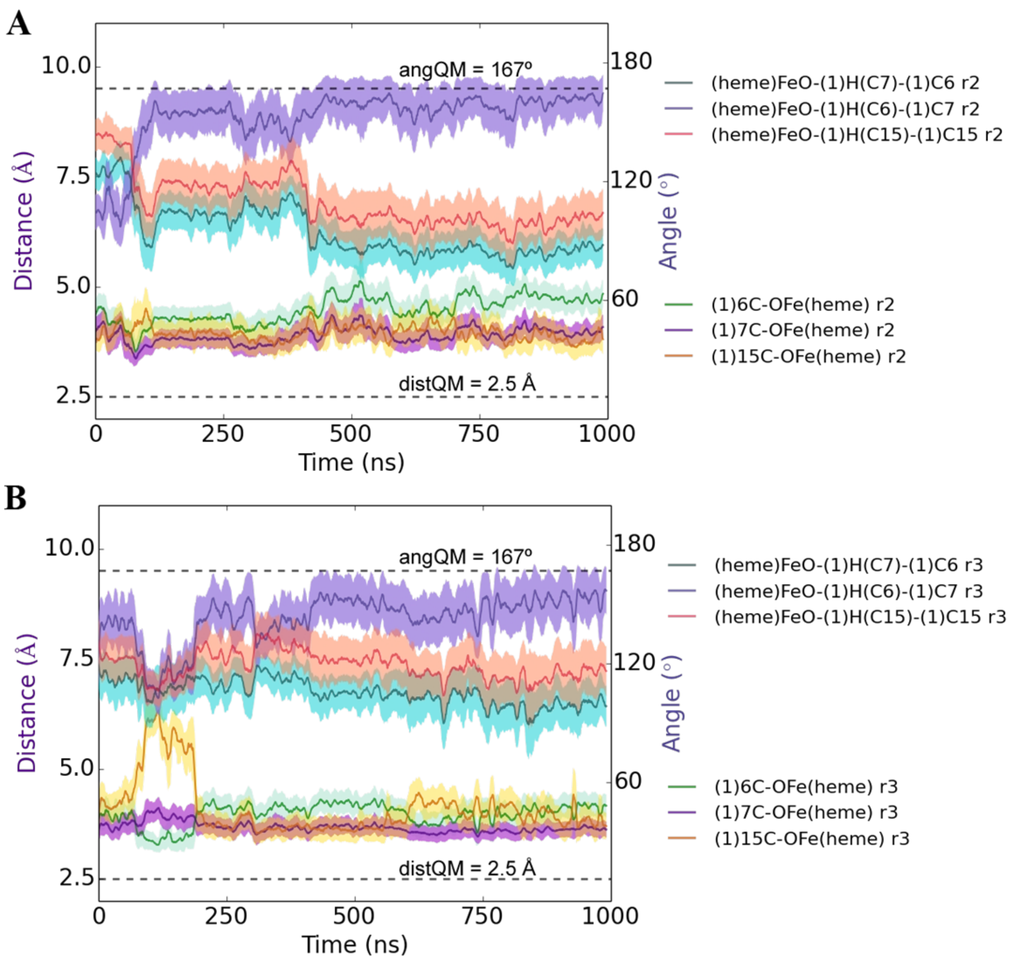

Figure S9. Plot of the C6,C7,C15(**1**)···OFe distances (y primary axis) and the O(Fe=O)–(**1**)-H(6C,7C,15C)–(**1**)-C(6,7,15) angles (y secondary axis) along the simulation time (x axis) of the second (A) and third (B) MD replica dataset.

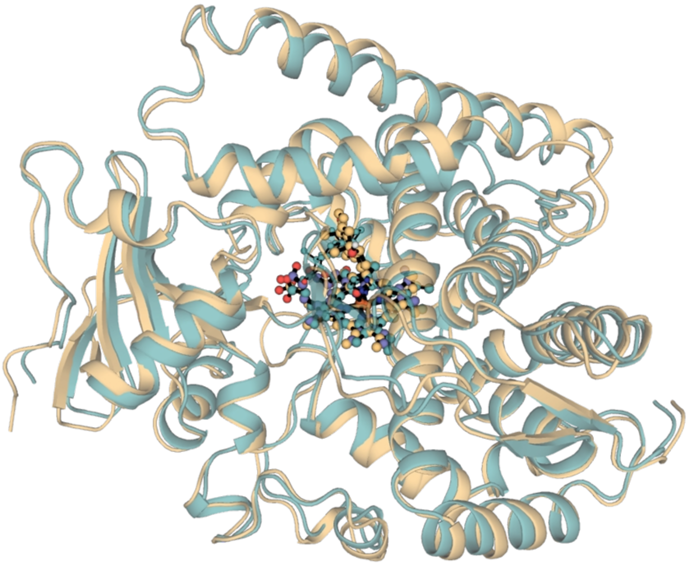

Figure S10. Overlay of MD representative conformation (light orange color) and available X-ray structure of LG-23 mutant (light teal color). Haem, substrate and imidazole are shown in ball and sticks.

HPLC choromatograms

A

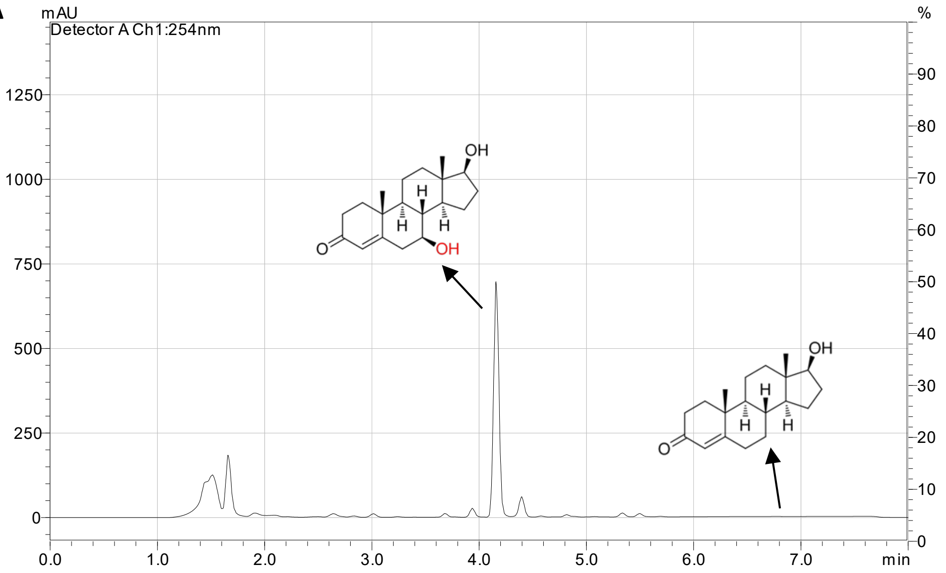

B

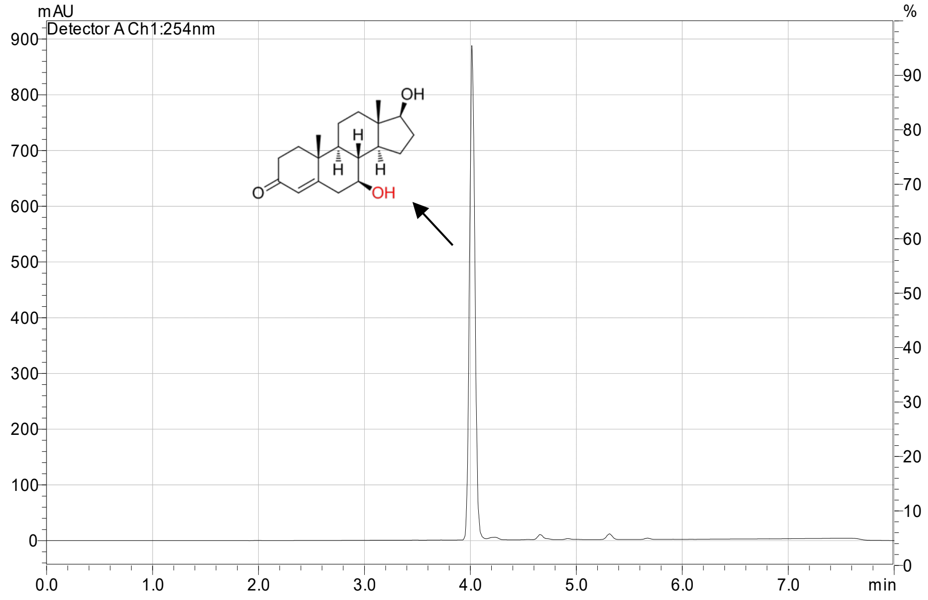

Figure S11. HPLC chromatograms of regio- and steroselective 7β-hydroxylation of testosterone (**1**) catalyzed by variant P450BM3 mutant LG-23: (A) reaction mixture; (B) product after purification.

A

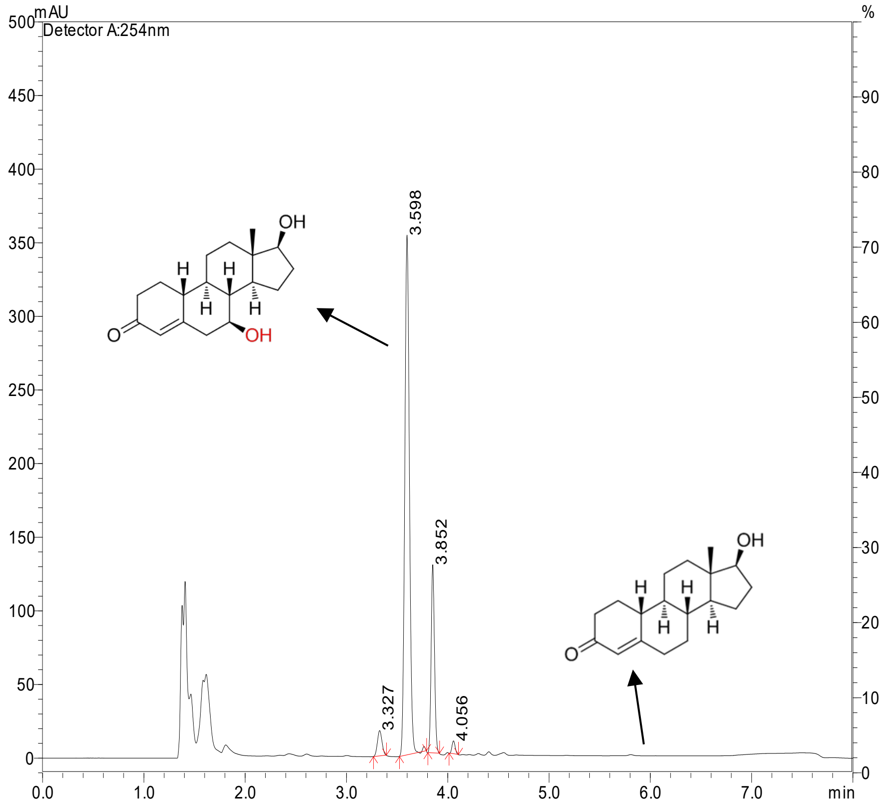

B

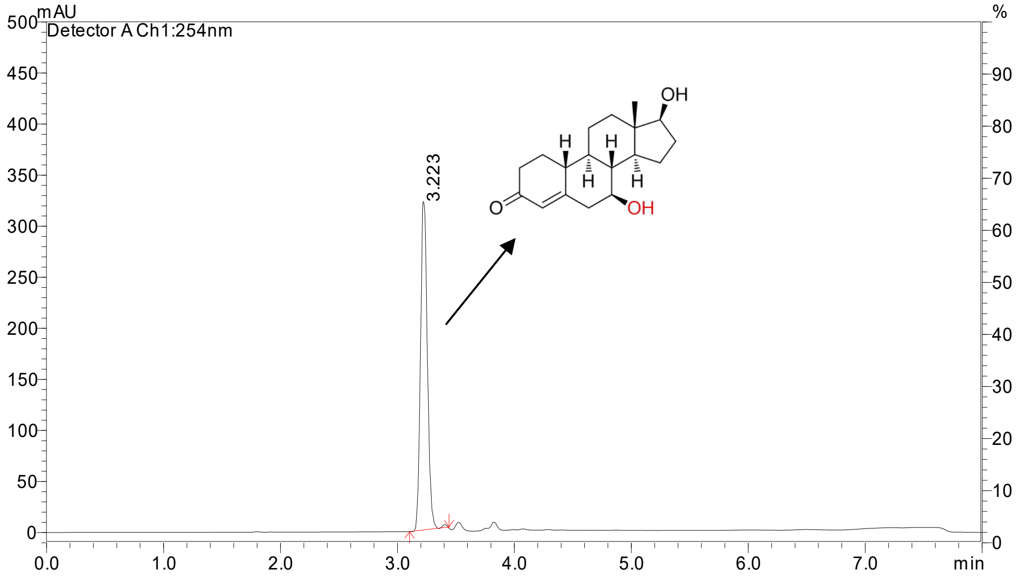

Figure S12. HPLC chromatograms of regio- and steroselective 7β-hydroxylation of nandrolone (**3**) catalyzed by variant P450BM3 mutant LG-23: (A) reaction mixture; (B) product after purification.

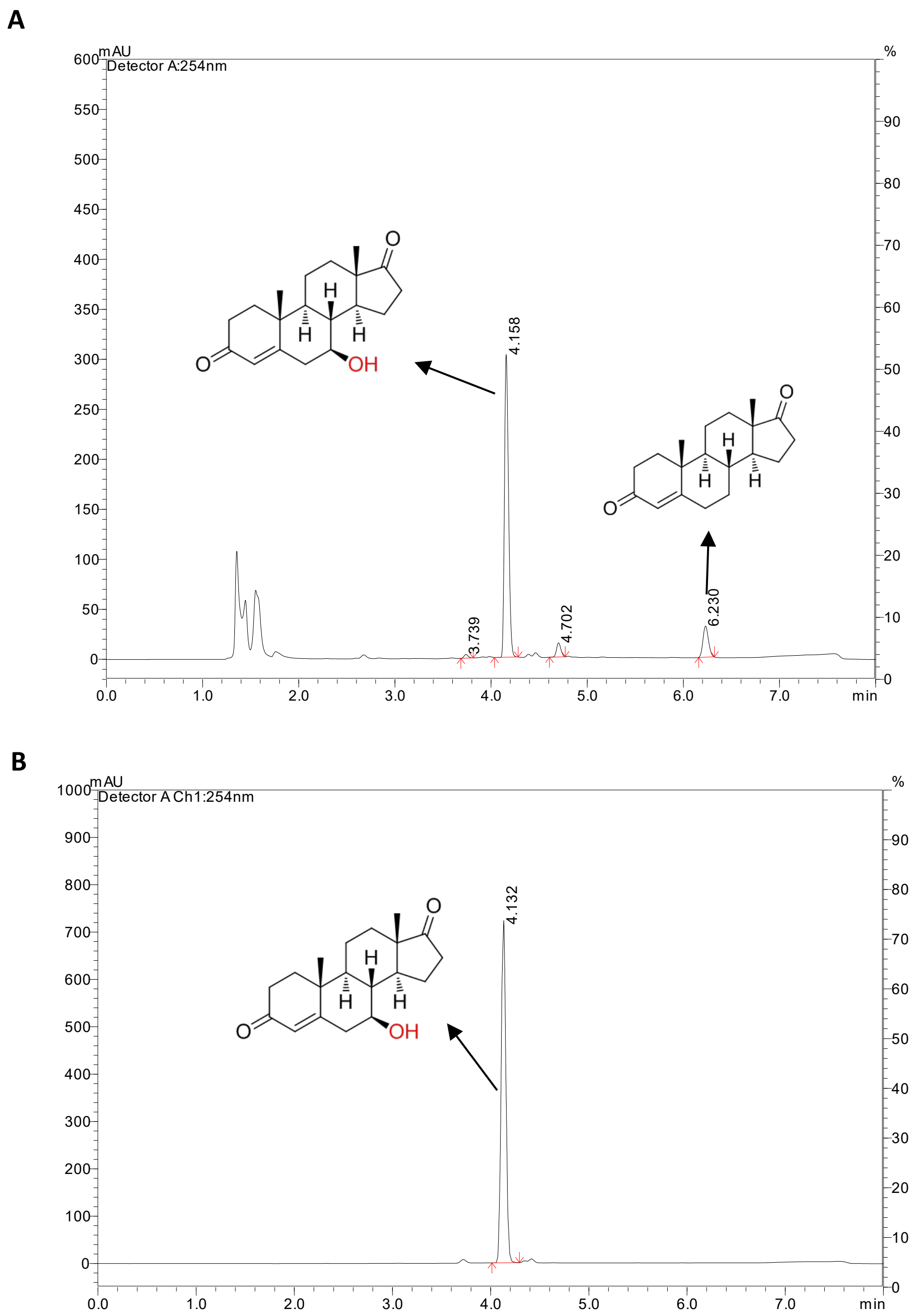

Figure S13. HPLC chromatograms of regio- and steroselective 7β-hydroxylation of 4-androstenedione **(5)** catalyzed by variant P450BM3 mutant LG-23: (A) reaction mixture; (B) product after purification.

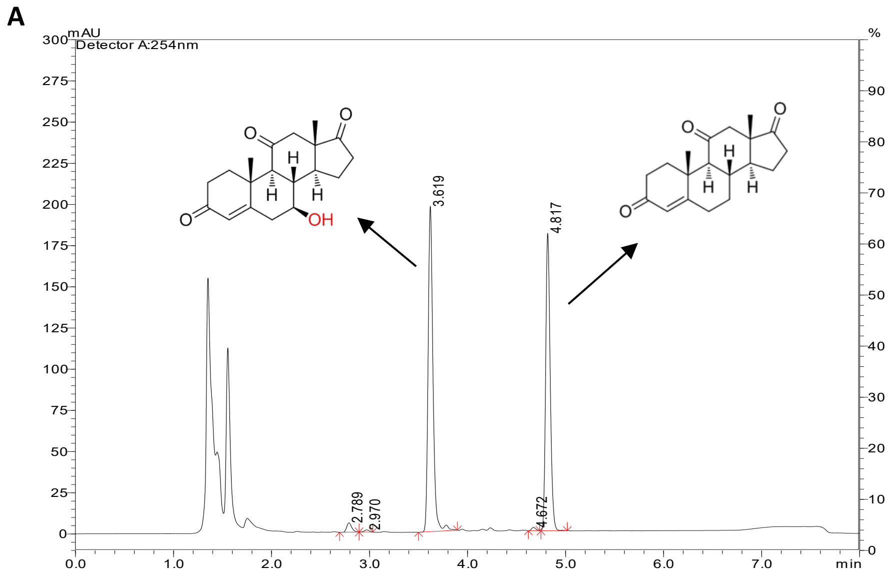

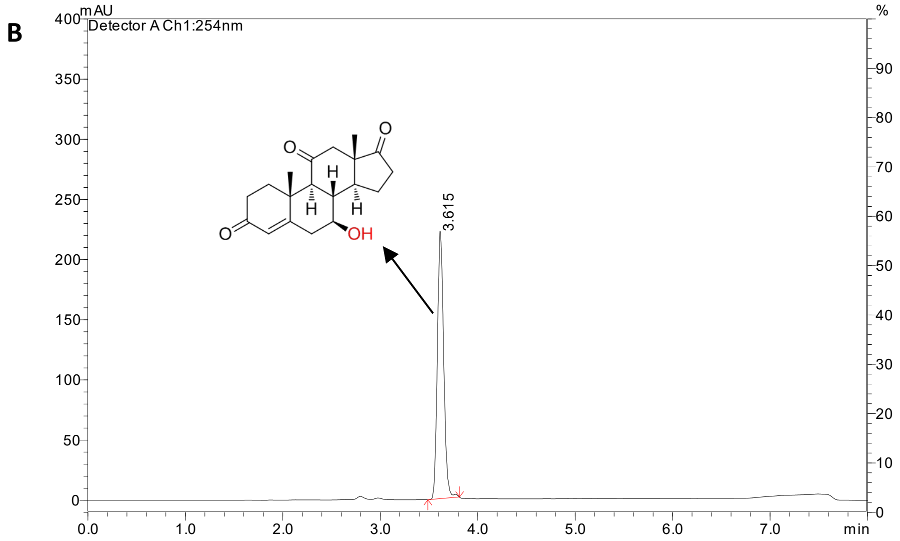

Figure S14. HPLC chromatograms of regio- and steroselective 7β-hydroxylation of adrenosterone (**7**) catalyzed by variant P450BM3 mutant LG-23: (A) reaction mixture; (B) product after purification.

A

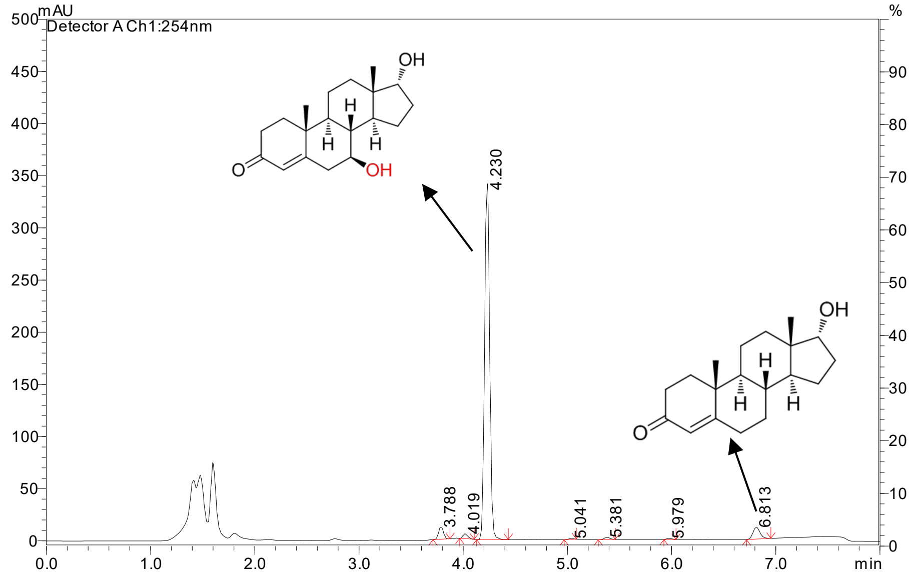

B

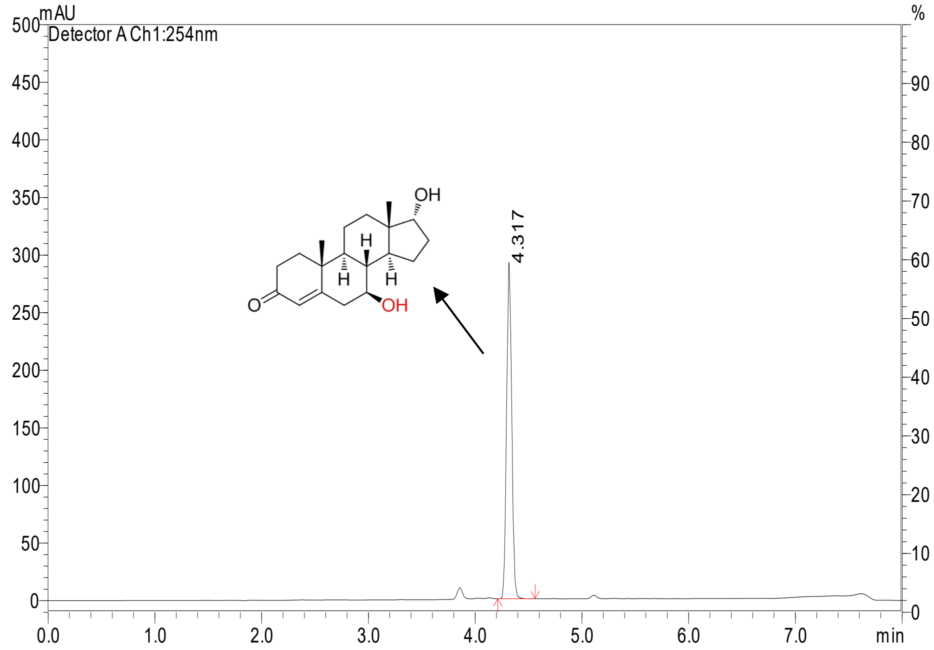

Figure S15. HPLC chromatograms of regio- and steroselective 7β-hydroxylation of epitestosterone (**9**) catalyzed by variant P450BM3 mutant LG-23: (A) reaction mixture; (B) product after purification.

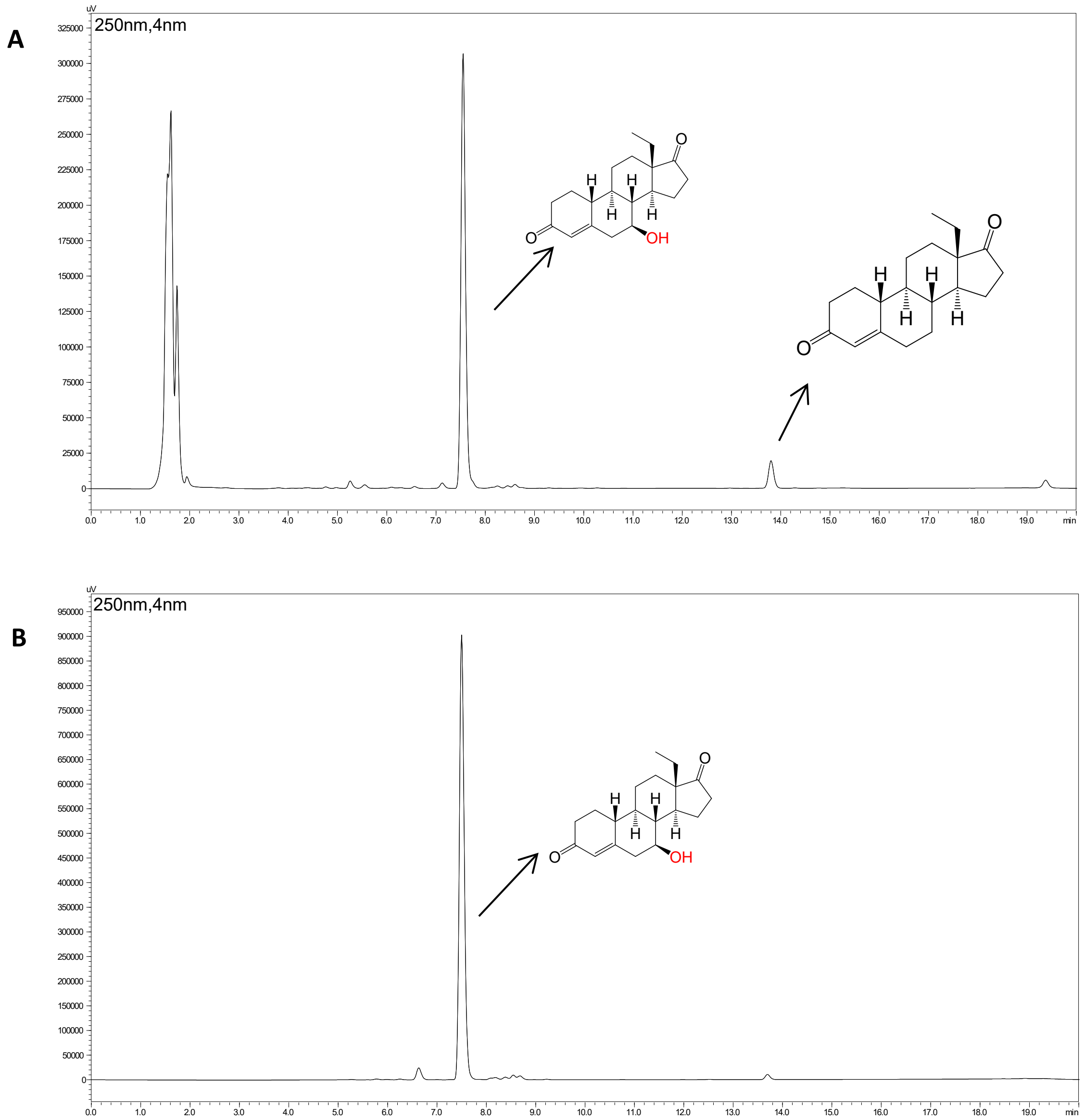

Figure S16. HPLC chromatograms of regio- and steroselective 7β-hydroxylation of 13-Ethylgon-4-ene-3,17-dione (**11**) catalyzed by variant P450BM3 mutant LG-23: (A) reaction mixture; (B) product after purification.

LC-MS spectrums

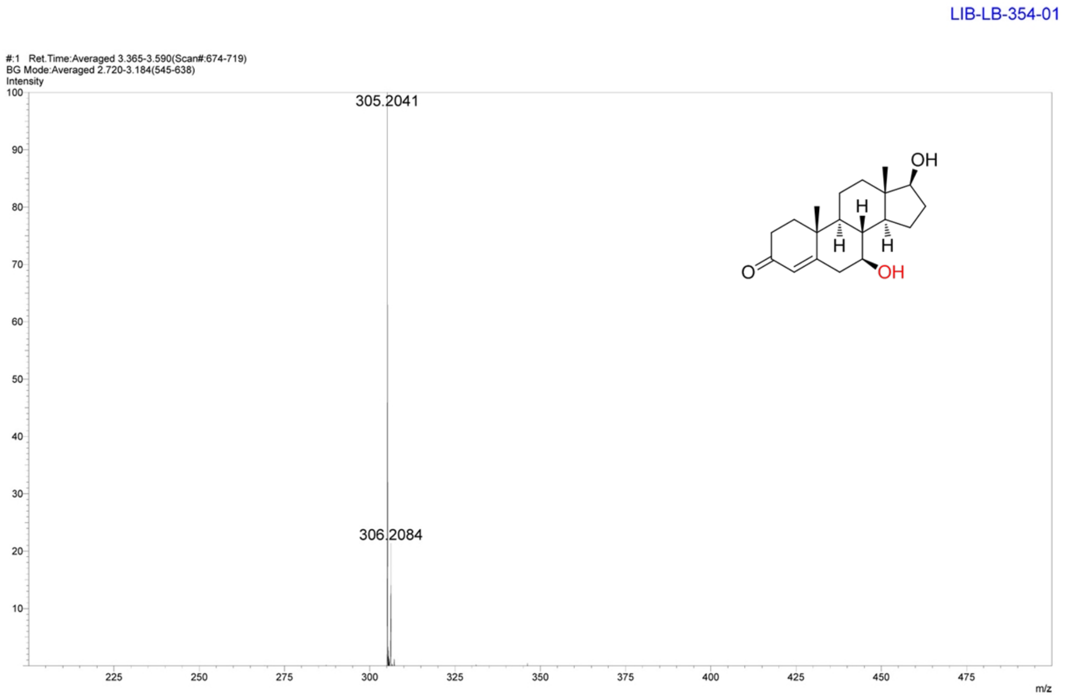

Figure S17. Mass Spectrum analysis for 7β-Hydroxytestosterone **(2)**.

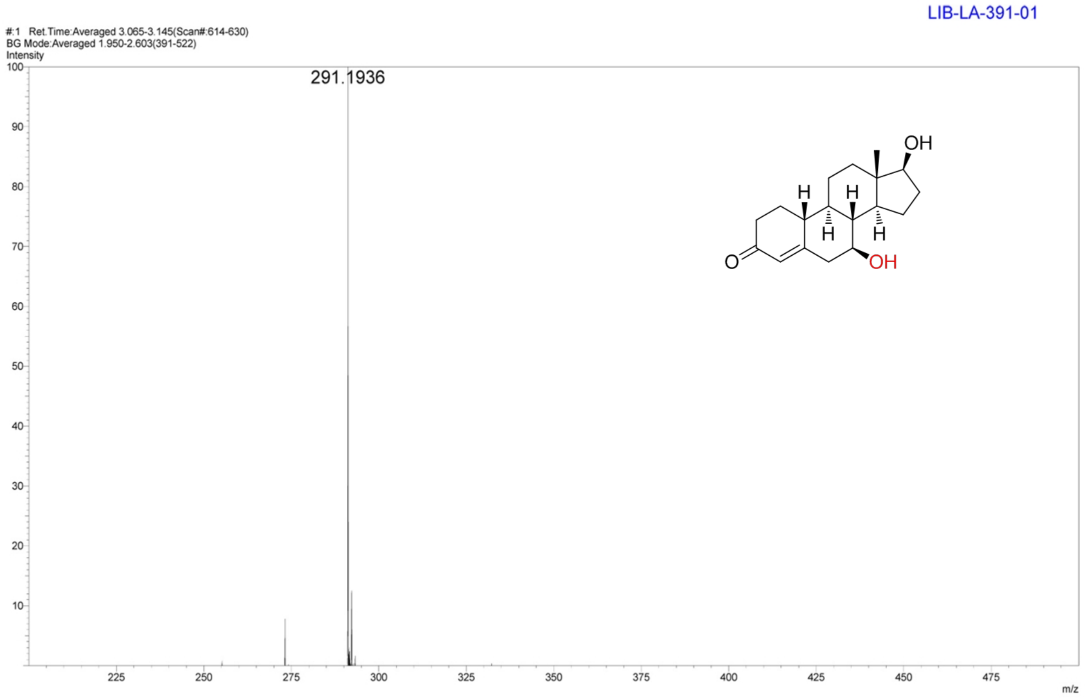

Figure S18. Mass Spectrum analysis for 7β-Hydroxynandrolone (**6**).

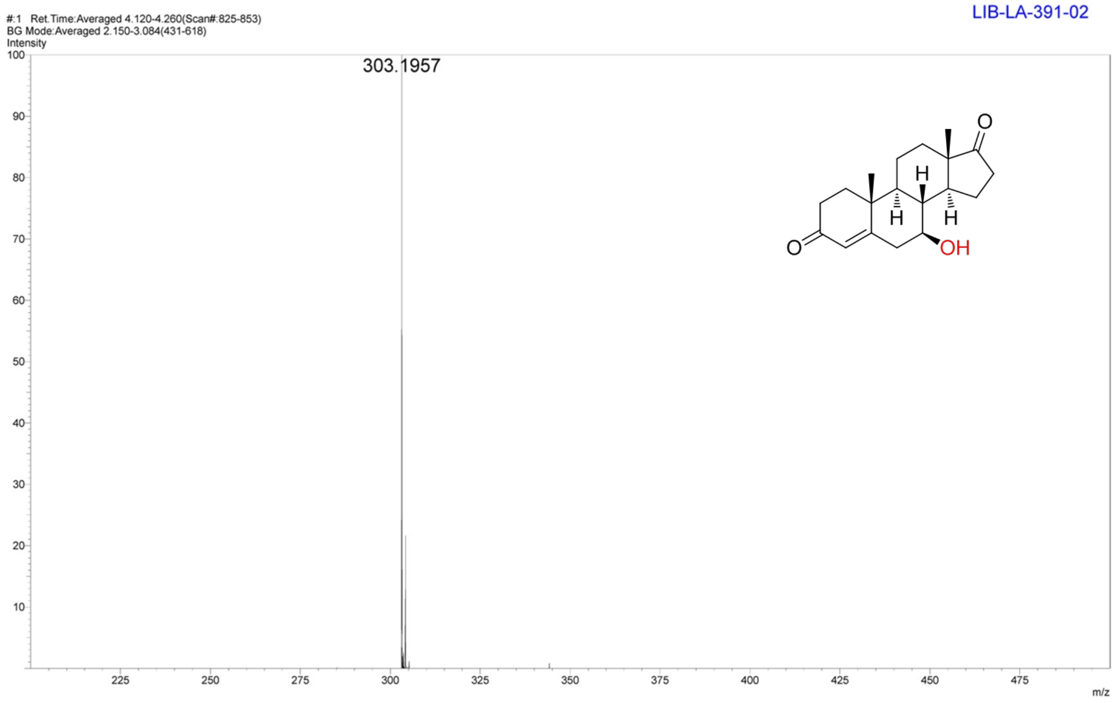

Figure S19. Mass Spectrum analysis for 7β-Hydroxy-4-androstenedione (**8**).

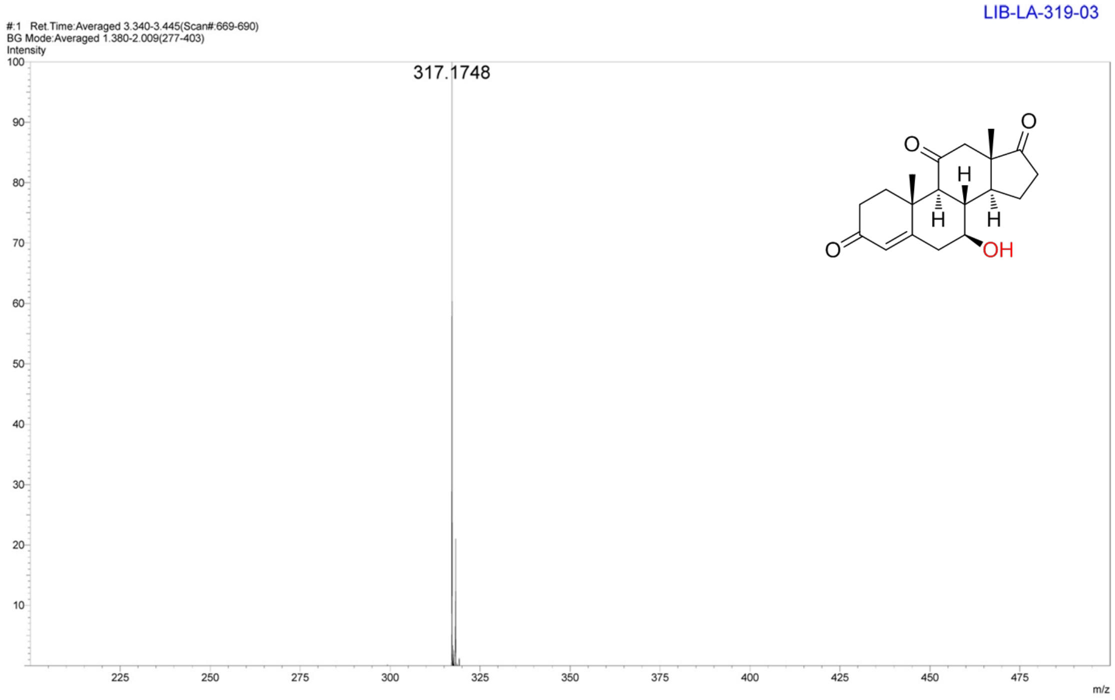

Figure S20. Mass Spectrum analysis for 7β-Hydroxyandrenosterone (**10**).

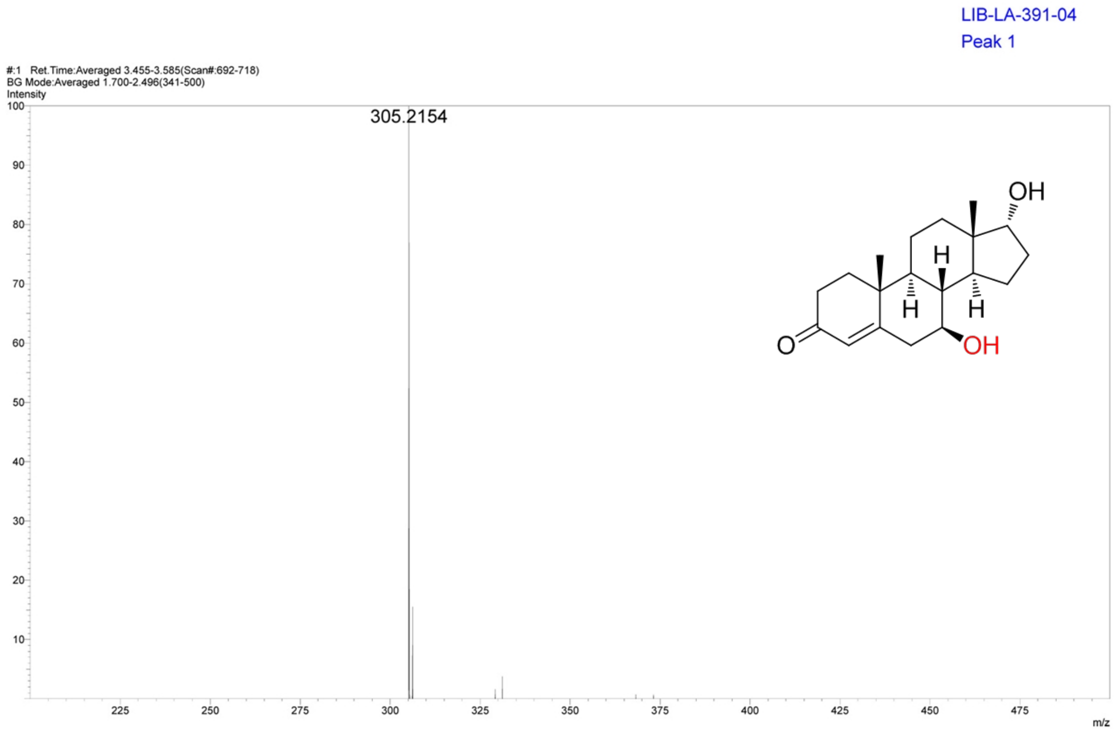

Figure S21. Mass Spectrum analysis for 7β-Hydroxyepitestosterone (**12**).

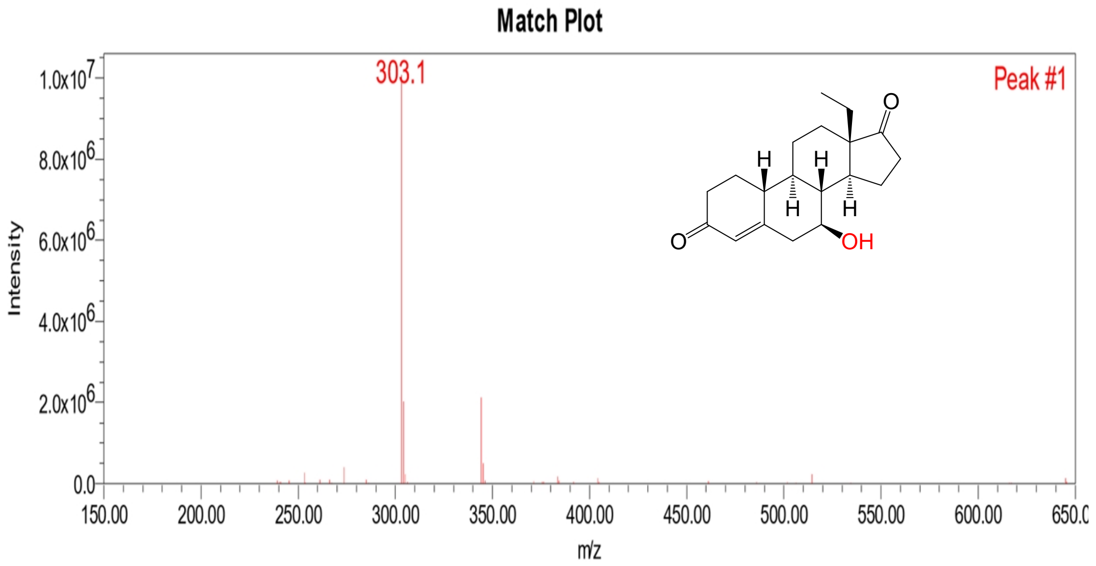

Figure S22. Mass Spectrum analysis for 7β-13-Ethylgon-4-ene-3,17-dion (**14**).

NMR Spectrums

Figure S23

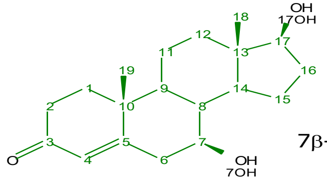

7β-Hydroxytestosterone

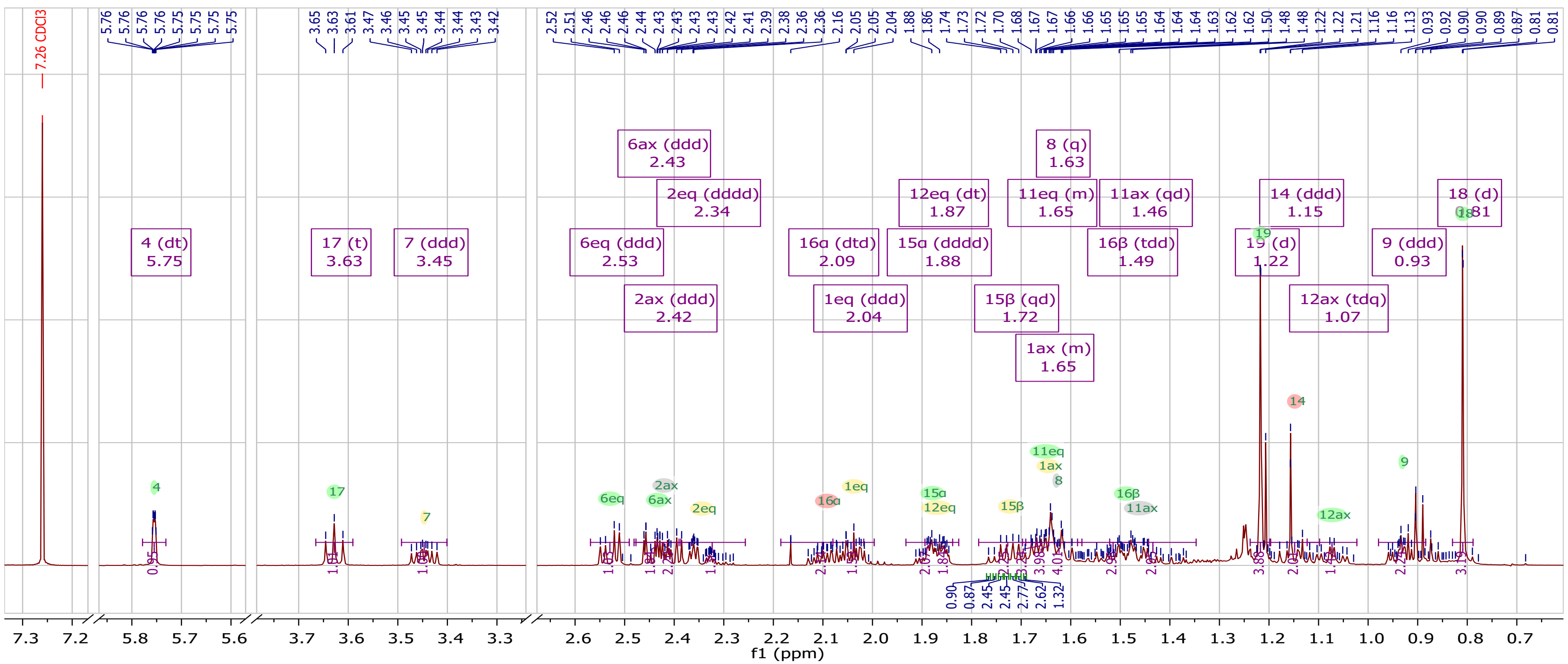

Figure S24

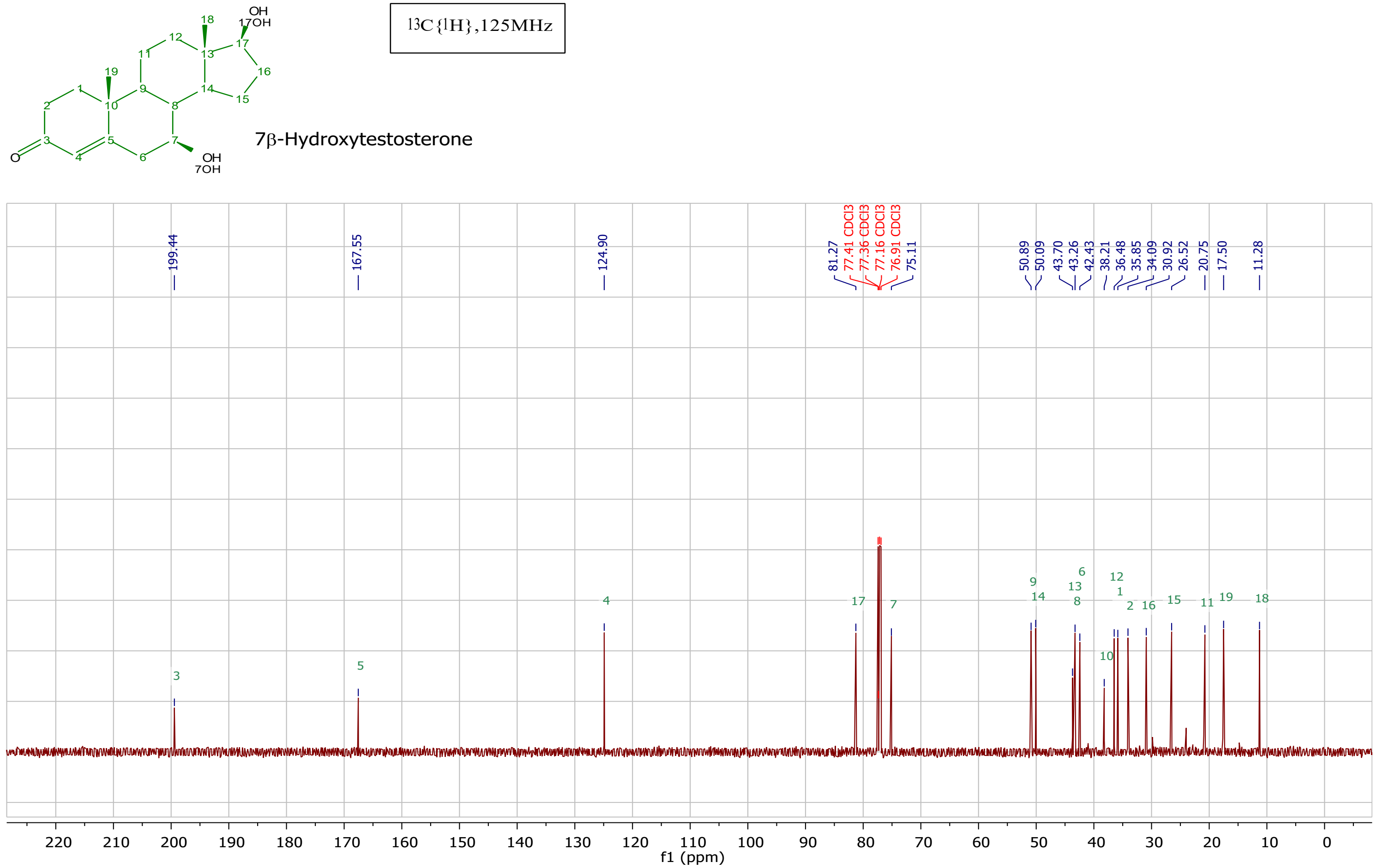
13C{1H},125MHz
7β-Hydroxytestosterone
17OH
7OH
199.44
167.55
124.90
81.27
77.41 CDCl3
77.36 CDCl3
77.16 CDCl3
76.91 CDCl3
75.11
50.89
50.09
43.70
43.26
42.43
38.21
36.48
35.85
34.09
30.92
26.52
20.75
17.50
11.28
220 210 200 190 180 170 160 150 140 130 120 110 100 90 80 70 60 50 40 30 20 10 0
f1 (ppm)

Figure S25

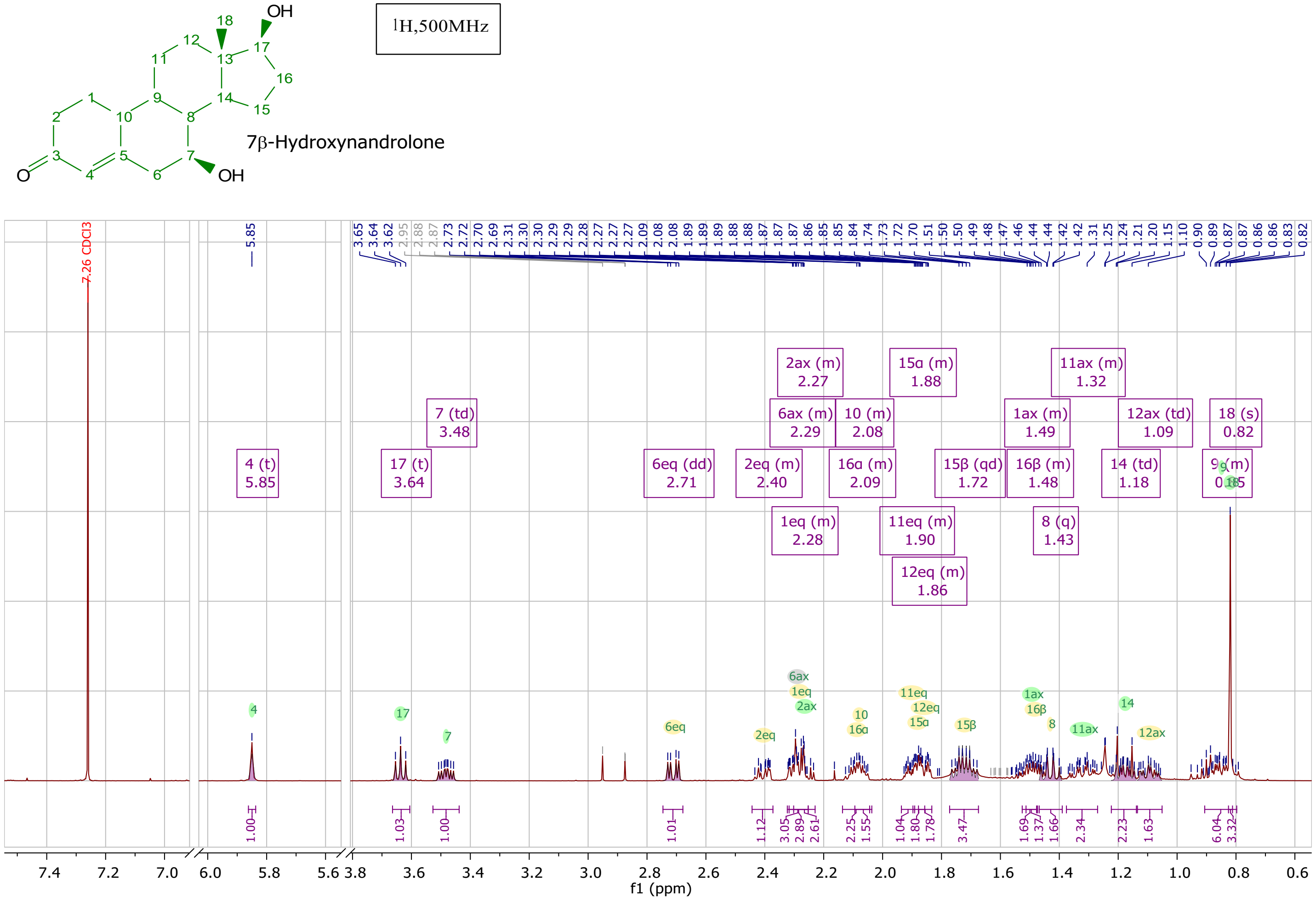
7β-Hydroxynandrolone
1H,500MHz
7.26 CDCl3
4 (t)
5.85
17 (t)
3.64
7 (td)
3.48
6eq (dd)
2.71
2eq (m)
2.40
2ax (m)
2.27
6ax (m)
2.29
1eq (m)
2.28
10 (m)
2.08
16α (m)
2.09
15α (m)
1.88
11eq (m)
1.90
12eq (m)
1.86
15β (qd)
1.72
1ax (m)
1.49
16β (m)
1.48
8 (q)
1.43
11ax (m)
1.32
14 (td)
1.18
12ax (td)
1.09
18 (s)
0.82
f1 (ppm)

Figure S26

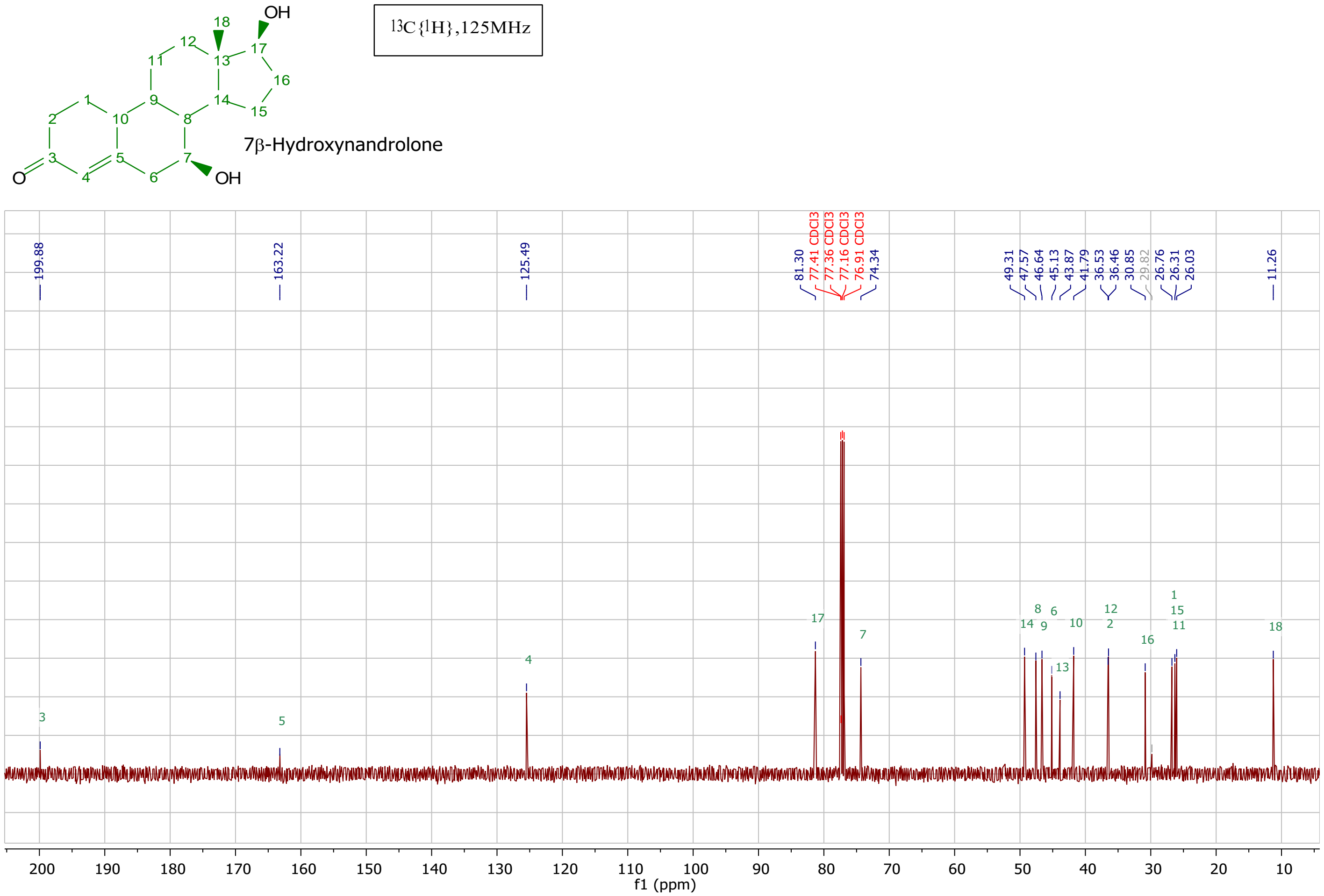
7β-Hydroxynandrolone
13C{1H},125MHz
199.88
163.22
125.49
81.30
77.41 CDCl3
77.36 CDCl3
77.16 CDCl3
76.91 CDCl3
74.34
49.31
47.57
46.64
45.13
43.87
41.79
36.53
36.46
30.85
29.82
26.76
26.31
26.03
11.26
f1 (ppm)

Figure S27

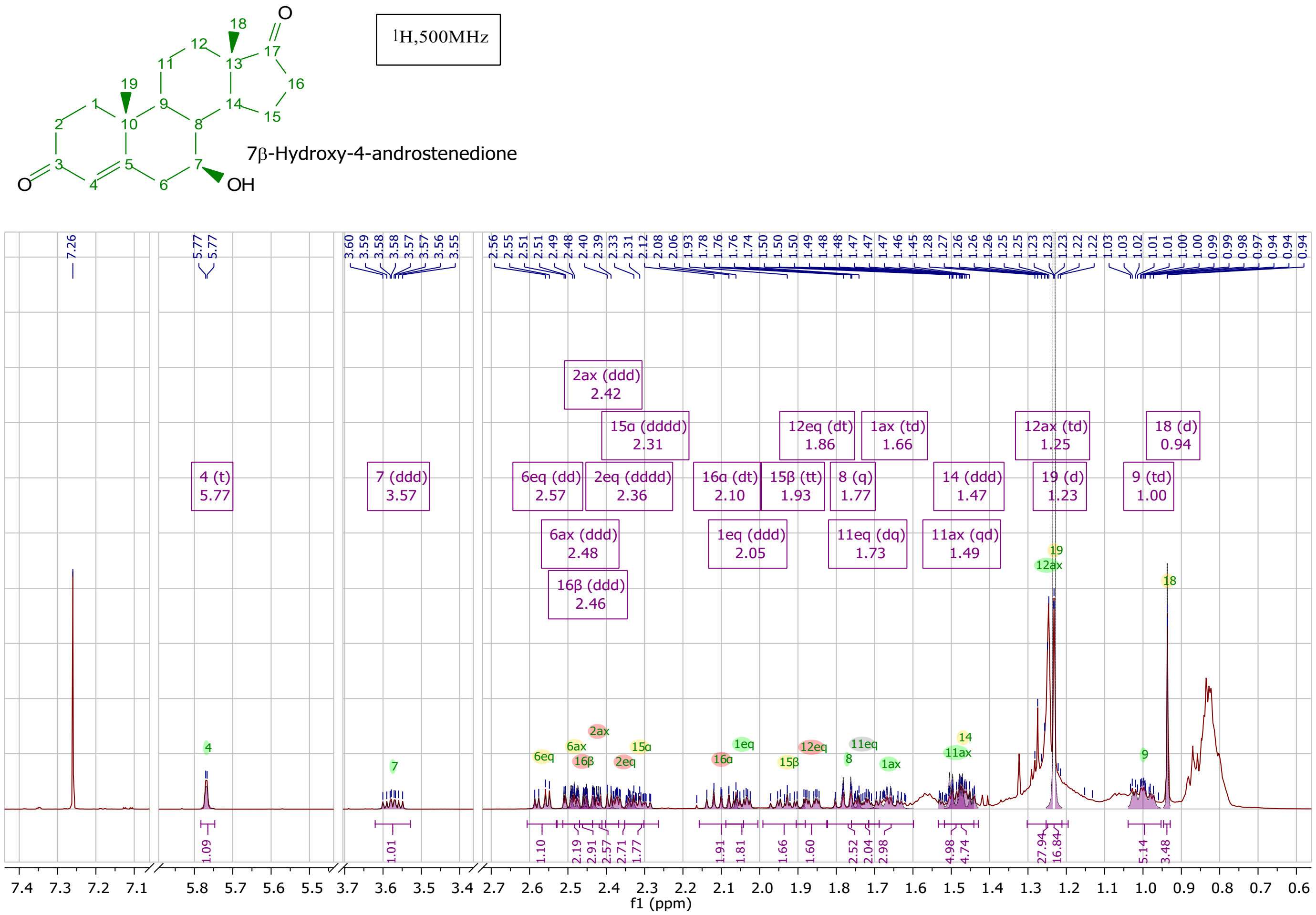

1H,500MHz
7β-Hydroxy-4-androstenedione
4 (t) 5.77
7 (ddd) 3.57
2ax (ddd) 2.42
15α (dddd) 2.31
6eq (dd) 2.57
2eq (dddd) 2.36
6ax (ddd) 2.48
16β (ddd) 2.46
16α (dt) 2.10
1eq (ddd) 2.05
12eq (dt) 1.86
15β (tt) 1.93
8 (q) 1.77
11eq (dq) 1.73
1ax (td) 1.66
14 (ddd) 1.47
11ax (qd) 1.49
12ax (td) 1.25
19 (d) 1.23
18 (d) 0.94
9 (td) 1.00
f1 (ppm)

Figure S28

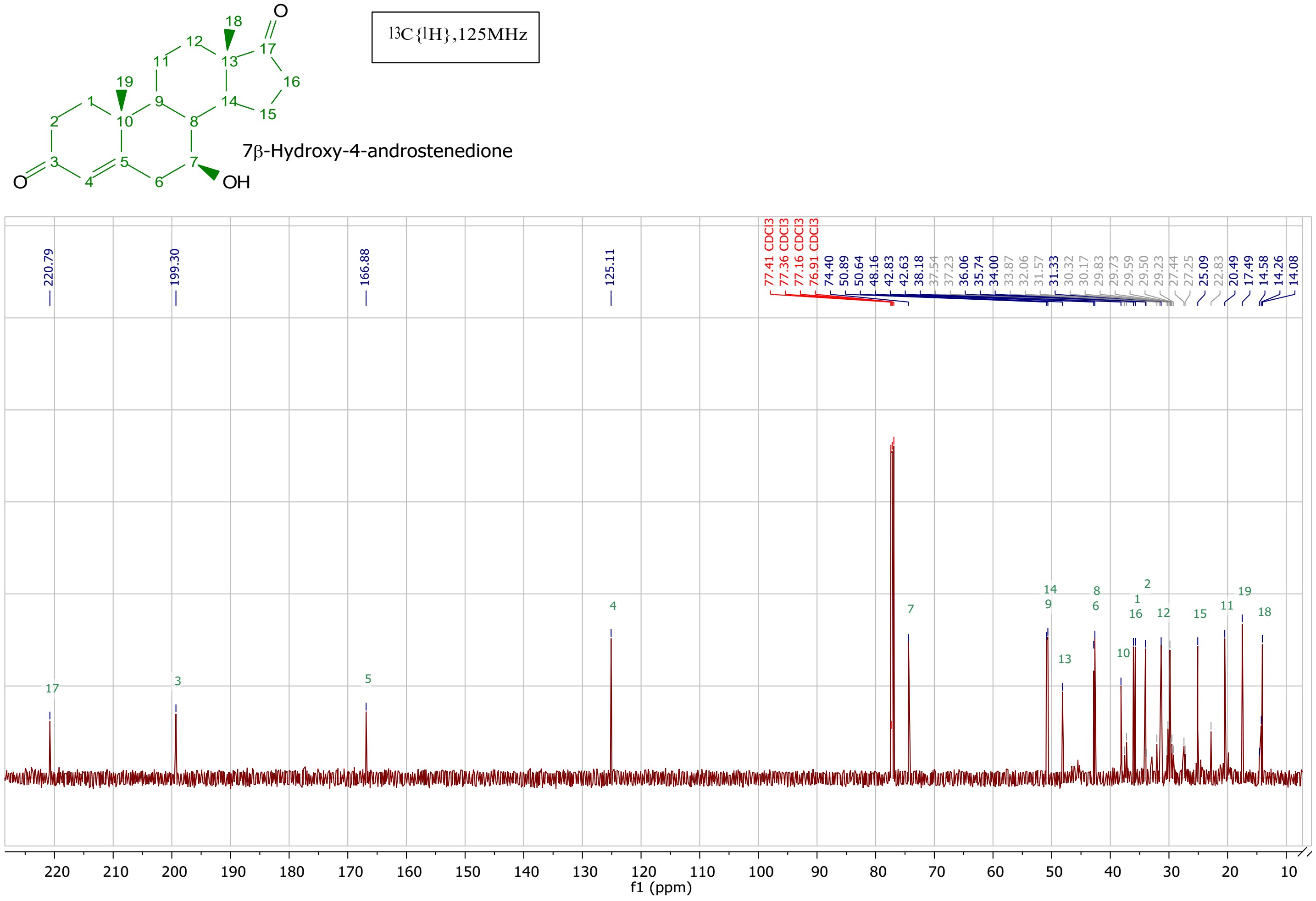
13C{1H},125MHz
7β-Hydroxy-4-androstenedione
220.79
199.30
166.88
125.11
77.41 CDCl3
77.36 CDCl3
77.16 CDCl3
76.91 CDCl3
74.40
50.89
50.64
48.16
42.83
42.63
38.18
37.54
37.23
36.06
35.74
34.00
33.87
32.06
31.57
31.33
30.32
30.17
29.83
29.73
29.59
29.50
29.23
27.44
27.25
25.09
22.83
20.49
17.49
14.58
14.26
14.08
f1 (ppm)

Figure S29

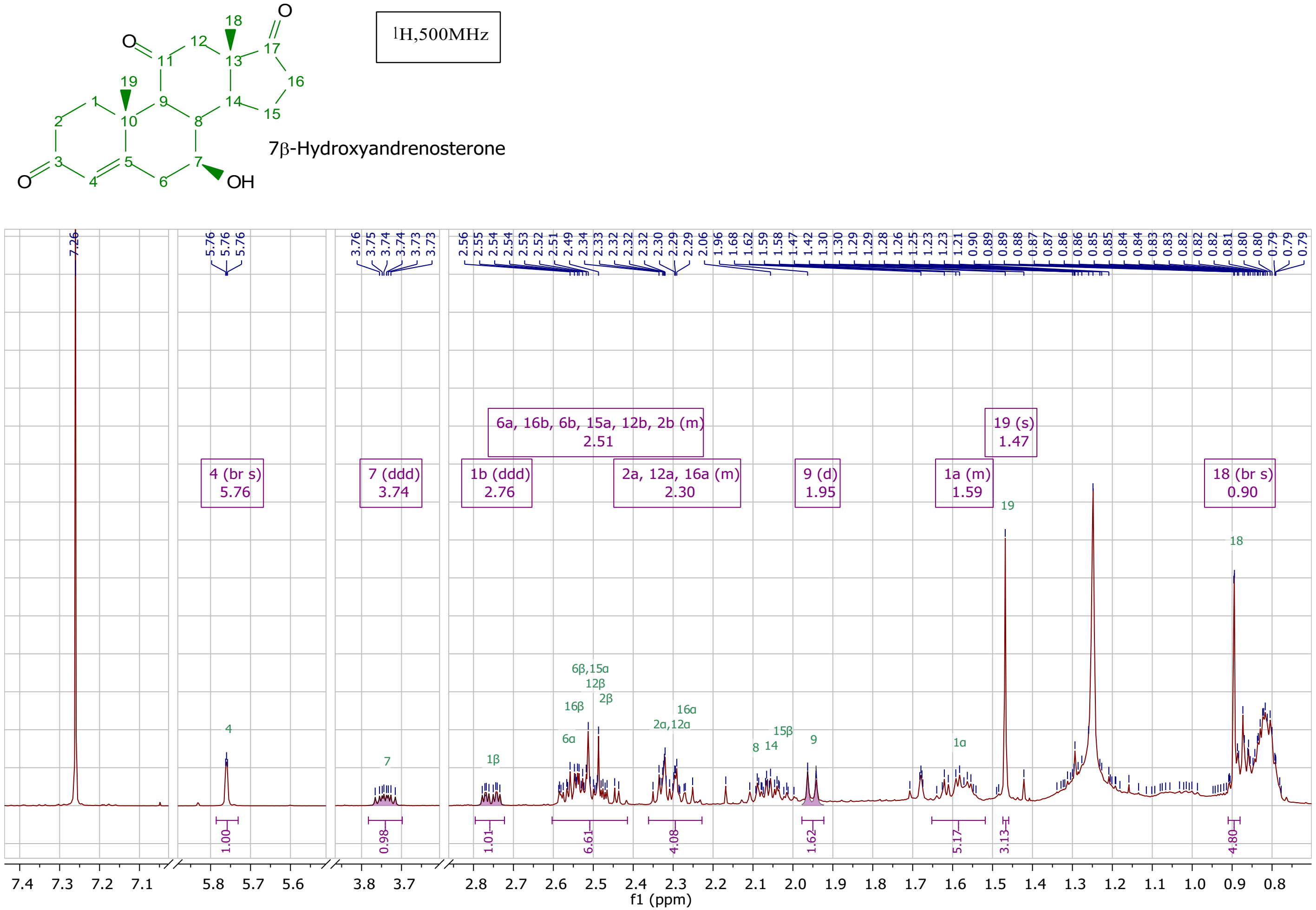

7β-Hydroxyandrenosterone
1H,500MHz
4 (br s) 5.76
7 (ddd) 3.74
1b (ddd) 2.76
6a, 16b, 6b, 15a, 12b, 2b (m) 2.51
2a, 12a, 16a (m) 2.30
9 (d) 1.95
1a (m) 1.59
19 (s) 1.47
18 (br s) 0.90
f1 (ppm)

Figure S30

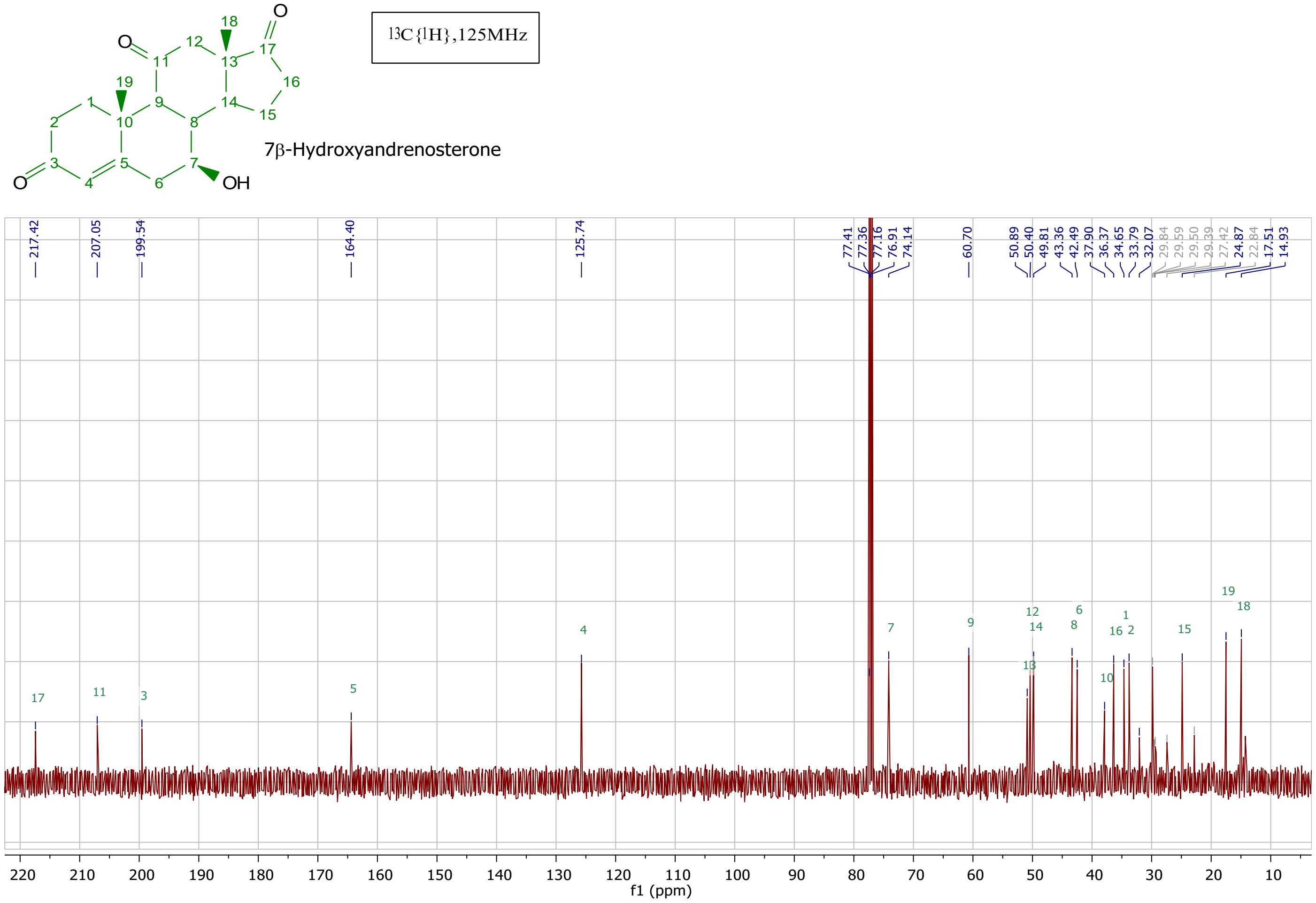

13C{1H},125MHz
7β-Hydroxyandrenosterone
217.42
207.05
199.54
164.40
125.74
77.41
77.36
77.16
76.91
74.14
60.70
50.89
50.40
49.81
43.36
42.49
37.90
36.37
34.65
33.79
32.07
29.84
29.59
29.50
29.39
27.42
24.87
22.84
17.51
14.93
f1 (ppm)

Figure S31

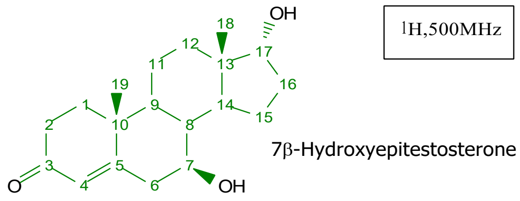
OH
18
12
17
11
13
16
19
1
9
14
2
10
8
15
3
5
7
O
4
6
OH

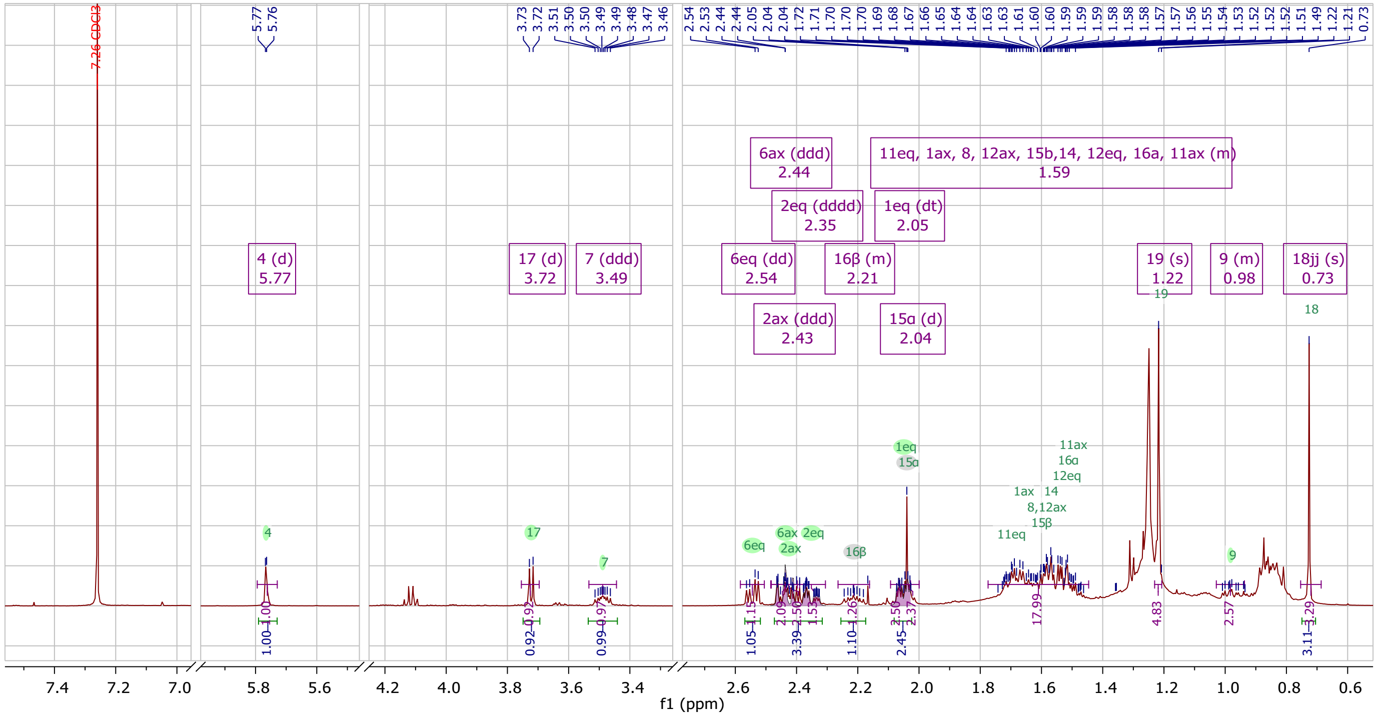
1H,500MHz
7β-Hydroxyepitestosterone
4 (d)
5.77
17 (d)
3.72
7 (ddd)
3.49
6eq (dd)
2.54
6ax (ddd)
2.44
2ax (ddd)
2.43
2eq (dddd)
2.35
16β (m)
2.21
1eq (dt)
2.05
15α (d)
2.04
11eq, 1ax, 8, 12ax, 15b,14, 12eq, 16a, 11ax (m)
1.59
19 (s)
1.22
9 (m)
0.98
18jj (s)
0.73
f1 (ppm)

Figure S32

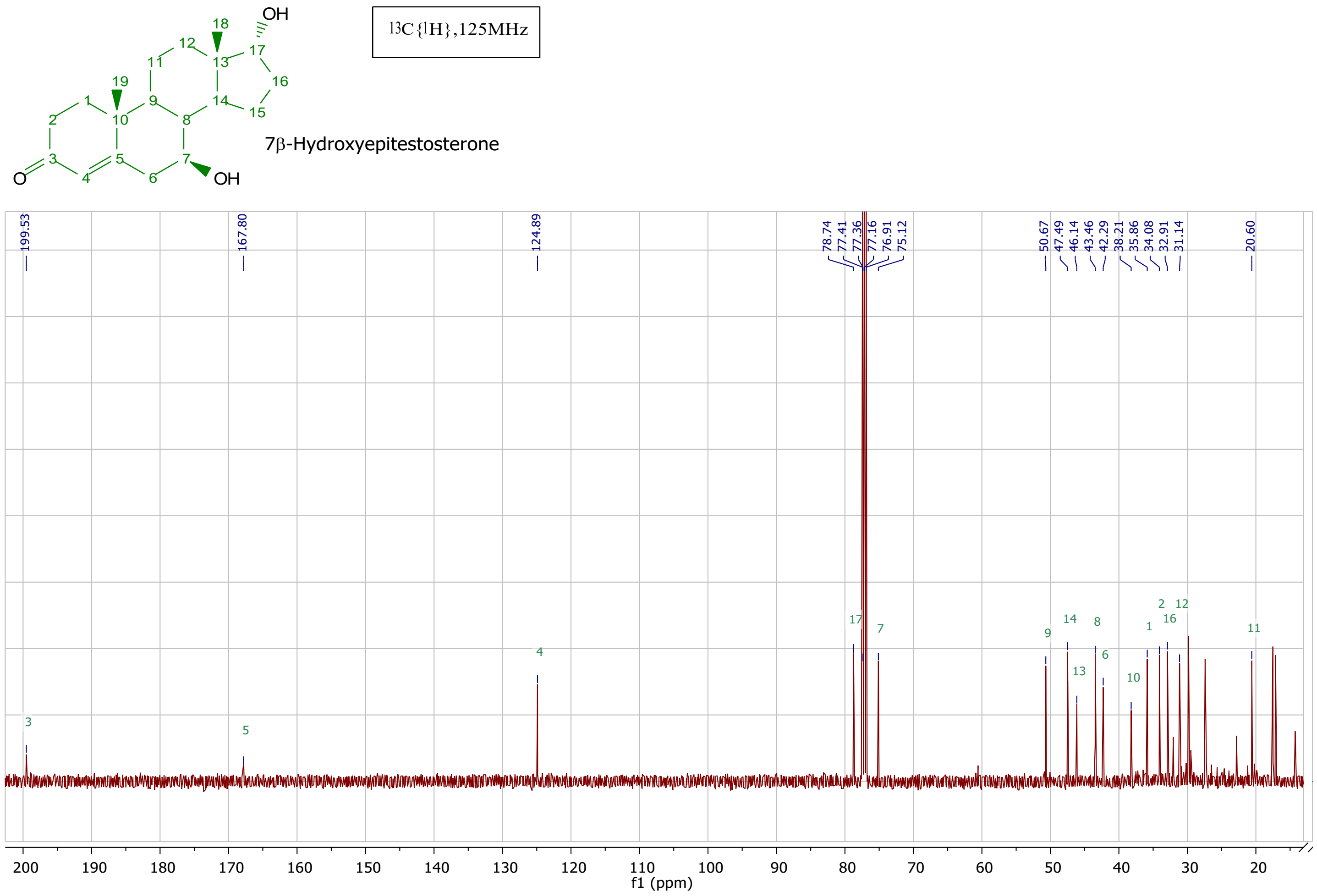
13C{1H},125MHz
7β-Hydroxyepitestosterone
199.53
167.80
124.89
78.74
77.41
77.36
77.16
76.91
75.12
50.67
47.49
46.14
43.46
42.29
38.21
35.86
34.08
32.91
31.14
20.60
f1 (ppm)

Figure S33

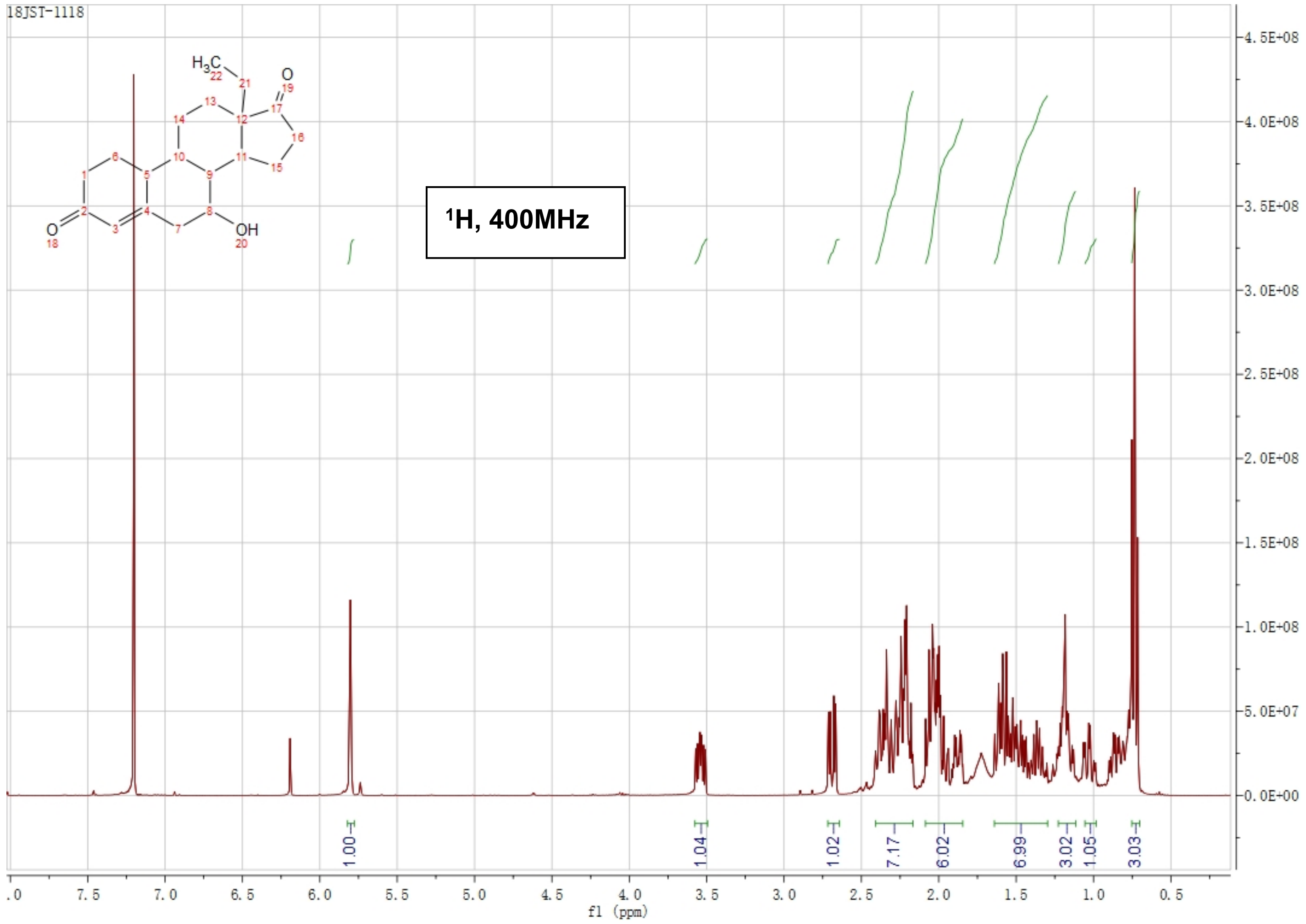
18JST-1118
^{1}H, 400MHz
H_3C
O
OH
1.00
1.04
1.02
7.17
6.02
6.99
3.02
1.05
3.03
f1 (ppm)

Figure S34

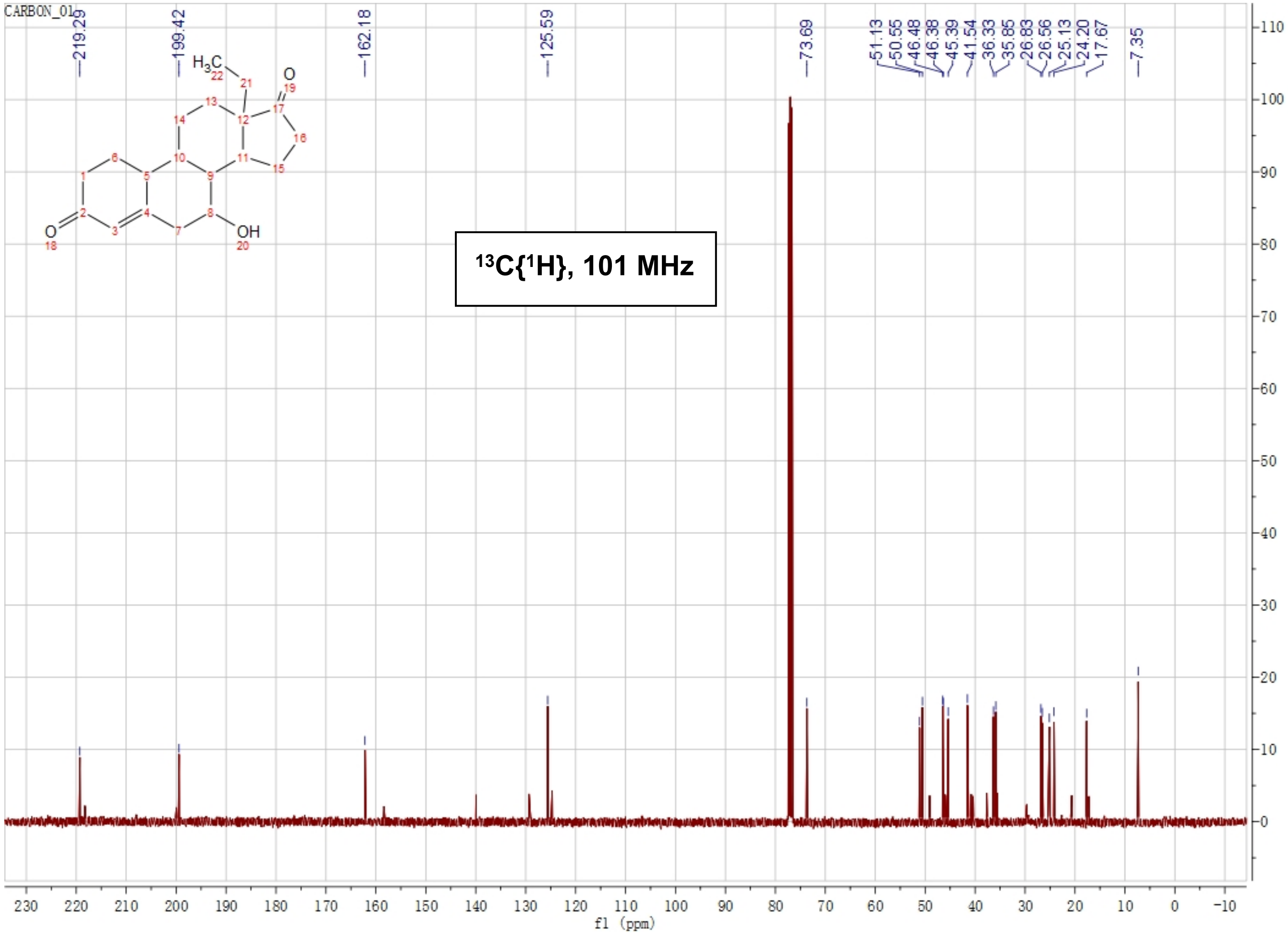
CARBON_01
219.29
199.42
162.18
125.59
73.69
51.13
50.55
46.48
46.38
45.39
41.54
36.33
35.85
26.83
26.56
25.13
24.20
17.67
7.35
$^{13}C\{^{1}H\}$, 101 MHz
f1 (ppm)

References

1. T. M. Jacobs, H. Yumerefendi, B. Kuhlman, A. Leaver-Fay, *Nucleic Acids Res*. **2015**, *43*, e34,
2. P. Emsley, B. Lohkamp, W. G. Scott, K. Cowtan. *Acta Crystallogr, D: Biol. Crystallogr.* **2010**, *66* (Pt 4), 486-501.
3. P. D. Adams, P. V. Afonine, G. Bunkoczi, V. B. Chen, I. W. Davis, N. Echols, J. J. Headd, L. W. Hung, G. J. Kapral, R. W. Grosse-Kunstleve, A. J. McCoy, N. W. Moriarty, R. Oeffner, R. J. Read, D. C. Richardson, J. S. Richardson, T. C. Terwilliger, P. H. Zwart, *Acta Crystallogr. D: Biol. Crystallogr.* **2010**, *66* (Pt 2), 213-221.
4. W. DeLano, *The PyMOL Molecular Graphics System. Version 0.99rc6 Schrödinger.* LLC, De Lano Scientific,: San Carlos, **2002**.
5. A. R. H. Narayan, G. Jiménez-Osés, P. Liu, S. Negretti, W. Zhao, M. M. Gilbert, R. O. Ramabhadran, Y.-F. Yang, L. R. Furan, Z. Li, L. M. Podust, J. Montgomery, K. N. Houk, D. H. Sherman, *Nat. Chem.* **2015,** *7*, 653–660.
6. M. J. Frisch, G. W. Trucks, H. B. Schlegel, G. E. Scuseria, M. A. Robb, J. R. Cheeseman, G. Scalmani, V. Barone, B. Mennucci, G. A. Petersson, H. Nakatsuji, M. Caricato, X. Li, H. P. Hratchian, A. F. Izmaylov, J. Bloino, G. Zheng, J. L. Sonnemberg, M. Hada, M. Ehara, K. Toyota, R. Fukuda, J. Hasegawa, M. Ishida, T. Nakajama, Y. Honda, O. Kitao, H. Nakai, T. Vreven, J. A. Montgomery Jr., J. E. Peralta, F. Ogliaro, M. Bearpark, J. J. Heyd, E. Brothers, K. N. Kudin, V. N. Staroverov, T. Keith, R. Kobayashi, J. Normand, K. Raghavachari, A. Rendell, J. C. Burant, S. S. Iyengar, J. Tomasi, M. Cossi, N. Rega, J. M. Millam, M. Klene, J. E. Knox, J. B. Cross, V. Bakken, C. Adamo, J. Jaramillo, R. Gomperts, R. E. Stratmann, O. Yazyev, A. J. Austin, R. Cammi, C. Pomelli, J. W. Ochtersky, R. L. Martin, K. Morokuma, V. G. Zakrzewski, G. A. Voth, P. Salvador, J. J. Dannanberg, S. Dapprich, A. D. Daniels, O. Farkas, J. B. Foresman, J. V. Ortiz, J. Cioslowski, D. J. Fox, GAUSSIAN09, Inc. Wallingford CT, **2013**.
7. C. Lee, W. Yang, R. G. Parr, *Phys. Rev. B* **1988**, 37, 785–789.
8. A. D. Becke, *J. Chem. Phys.* **1993**, *98*, 5648–5652.
9. P. J. Hay, W. R. Wadt, *J. Chem. Phys.* **1985**, *82*, 270–283.
10. S. Grimme, J. Antony, S. Ehrlich, H. Krieg, *J. Chem. Phys.* **2010**, *132*, 154104.
11. D. A. Case, T. A. Darden, T. E. Cheatham, C. L. Simmerling, J. Wang, R. E. Duke, R. Luo, M. Crowley, R. C. Walker, W. Zhang, K. M. Merz, B. Wang, S. Hayik, A. Roitberg, G. Seabra, I. Kolossváry, K. F. Wong, F. Paesani, J. Vanicek, X. Wu, S. R. Brozell, T. Steinbrecher, H. Gohlke, L. Yang, C. Tan, J. Mongan, V. Hornak, G. Cui, D. H. Mathews, M. G. Seetin, C. Sagui, V. Babin, P. A. Kollman, AMBER 16, University of California, San Francisco, **2016**.
12. J. Wang, R. M. Wolf, J. W. Caldwell, P. A. Kollman, D. A. Case, *J. Comp. Chem*. **2004**, *25*, 1157-1174.
13. C. I. Bayly, P. Cieplak, W. Cornell, P. A. Kollman, *J. Phys. Chem*. **1993,** *97,* 10269- 10280.
14. B. H. Besler, K. M. Merz, P. A. Kollman, *J. Comp. Chem*. **1990**, *11*, 431-439.
15. U. C. Singh, P. A. Kollman, *J. Comp. Chem*. **1984**, *5*, 129-145.
16. K. Shahrokh, A. Orendt, G. S. Yost, T. E. Cheatham, *J. Comput. Chem.* **2012**, *33*, 119–133.
17. R. Anandakrishnan, B. Aguilar, A. V. Onufriev, *Nucleic Acids Res*. **2012,** *40*, W537- W541.
18. W. L. Jorgensen, J. Chandrasekhar, J. D. Madura, R. W. Impey, M. L. Klein, *J. Chem. Phys*. **1983**, *79*, 926-935.

19. K. Lindorff-Larsen, S. Piana, K. Palmo, P. Maragakis, J.L. Klepeis, R.O. Dror, D. E. Shaw, *Proteins* **2010**, *78*, 1950-1958.
20. H. Li, T. L. Poulos, *Nat. Struct. Biol*. **1997,** *4*, 140-146.
21. I. W. Davis, A W. B. rendall III, D. C. Richardson, J. S. Richardson, *Structure* **2006,** *14*, 265-274.
22. E. L. Humphris, T. Kortemme, *Structure* **2008,** *16*, 1777-1788.
23. C. A. Smith, T. Kortemme, *J. Mol. Biol*. **2008**, *380*, 742-756.
24. T. Darden, D. York, L. Pedersen, *J. Chem. Phys*. **1993**, *98*, 10089-10092.
25. S. Kille, F. E. Zilly, J. P. Acevedo, M. T. Reetz, *Nature Chem*., **2011**, *3*, 738-748.
